# Neoadjuvant therapy *versus* upfront surgery in resectable pancreatic cancer: reconstructed patient-level meta-analysis of randomized clinical trials

**DOI:** 10.1093/bjsopen/zrae087

**Published:** 2024-09-27

**Authors:** Daniel Aliseda, Pablo Martí-Cruchaga, Gabriel Zozaya, Nuria Blanco, Mariano Ponz, Ana Chopitea, Javier Rodríguez, Eduardo Castañón, Fernando Pardo, Fernando Rotellar

**Affiliations:** HPB and Liver Transplant Unit, Department of General Surgery, Clinica Universidad de Navarra, University of Navarra, Pamplona, Spain; HPB and Liver Transplant Unit, Department of General Surgery, Clinica Universidad de Navarra, University of Navarra, Pamplona, Spain; Institute of Health Research of Navarra (IdisNA), Pamplona, Spain; HPB and Liver Transplant Unit, Department of General Surgery, Clinica Universidad de Navarra, University of Navarra, Pamplona, Spain; Institute of Health Research of Navarra (IdisNA), Pamplona, Spain; HPB and Liver Transplant Unit, Department of General Surgery, Clinica Universidad de Navarra, University of Navarra, Pamplona, Spain; Institute of Health Research of Navarra (IdisNA), Pamplona, Spain; Institute of Health Research of Navarra (IdisNA), Pamplona, Spain; Department of Oncology, Clinica Universidad de Navarra, University of Navarra, Pamplona, Spain; Institute of Health Research of Navarra (IdisNA), Pamplona, Spain; Department of Oncology, Clinica Universidad de Navarra, University of Navarra, Pamplona, Spain; Institute of Health Research of Navarra (IdisNA), Pamplona, Spain; Department of Oncology, Clinica Universidad de Navarra, University of Navarra, Pamplona, Spain; Institute of Health Research of Navarra (IdisNA), Pamplona, Spain; Department of Oncology, Clinica Universidad de Navarra, University of Navarra, Pamplona, Spain; HPB and Liver Transplant Unit, Department of General Surgery, Clinica Universidad de Navarra, University of Navarra, Pamplona, Spain; Institute of Health Research of Navarra (IdisNA), Pamplona, Spain; HPB and Liver Transplant Unit, Department of General Surgery, Clinica Universidad de Navarra, University of Navarra, Pamplona, Spain; Institute of Health Research of Navarra (IdisNA), Pamplona, Spain

## Abstract

**Background:**

Neoadjuvant treatment has shown promising results in patients with borderline resectable pancreatic ductal adenocarcinoma. The potential benefits of neoadjuvant treatment on long-term overall survival in patients with resectable pancreatic ductal adenocarcinoma have not yet been established. The aim of this study was to compare long-term overall survival of patients with resectable pancreatic ductal adenocarcinoma based on whether they received neoadjuvant treatment or underwent upfront surgery.

**Methods:**

A systematic review including randomized clinical trials on the overall survival outcomes between neoadjuvant treatment and upfront surgery in patients with resectable pancreatic ductal adenocarcinoma was conducted up to 1 August 2023 from PubMed, MEDLINE and Web of Science databases. Patient-level survival data was extracted and reconstructed from available Kaplan–Meier curves. A frequentist one-stage meta-analysis was employed, using Cox-based models and a non-parametric method (restricted mean survival time), to assess the difference in overall survival between groups. A Bayesian meta-analysis was also conducted.

**Results:**

Five randomized clinical trials comprising 625 patients were included. Among patients with resectable pancreatic ductal adenocarcinoma, neoadjuvant treatment was not significantly associated with a reduction in the hazard of death compared with upfront surgery (shared frailty HR 0.88, 95% c.i. 0.72 to 1.08, *P* = 0.223); this result was consistent in the non-parametric restricted mean survival time model (+2.41 months, 95% c.i. −1.22 to 6.04, *P* < 0.194), in the sensitivity analysis that excluded randomized clinical trials with a high risk of bias (shared frailty HR 0.91 (95% c.i. 0.72 to 1.15; *P* = 0.424)) and in the Bayesian analysis with a posterior shared frailty HR of 0.86 (95% c.i. 0.70 to 1.05).

**Conclusion:**

Neoadjuvant treatment does not demonstrate a survival advantage over upfront surgery for patients with resectable pancreatic ductal adenocarcinoma.

## Teaser

This study aimed to assess the impact of neoadjuvant treatment compared with upfront surgery on the long-term overall survival of patients with resectable pancreatic ductal adenocarcinoma. A systematic review and reconstructed patient-level survival data meta-analysis including five RCTs with 625 patients revealed that neoadjuvant treatment did not significantly reduce the hazard of death compared with upfront surgery in resectable pancreatic ductal adenocarcinoma patients, suggesting that neoadjuvant treatment does not provide a substantial survival advantage over upfront surgery for this patient population.

## Introduction

In the USA, the number of deaths over the next 10 years from pancreatic cancer will significantly increase to become the second leading cause of cancer-related death^1^. Upon diagnosis, 10–20% of patients with pancreatic ductal adenocarcinoma (PDAC) have the option of surgical treatment^2^. For these patients, complete tumour removal with clear margins is the only potentially curative approach^3^. The updated guidelines now support neoadjuvant treatment (NAT) for patients with borderline resectable PDAC (BR-PDAC). In these patients, NAT increases the likelihood of achieving oncologically adequate resections, enabling tumour removal with free-tumour margins. Additionally, NAT allows for better identification of biologically aggressive tumours, helping to identify patients who may benefit the most from surgery^4^. These advantages are further enhanced by the guarantee of administering systemic therapy irrespective of postoperative complications or early recurrence.

The potential advantages of NAT in patients with BR-PDAC may be applicable to those with resectable PDAC (R-PDAC). However, evidence supporting NAT use in patients with R-PDAC is limited and derived mostly from retrospective studies with inherent biases^5^. Hence, the current standard of care for R-PDAC is upfront surgery (US) followed by adjuvant chemotherapy (CMT)^3^.

Despite well designed randomized clinical trials (RCTs), results regarding NAT in R-PDAC have been controversial. Studies have shown favourable survival outcomes following NAT, while others found no significant difference compared with US^4,6^. Attempts to pool survival outcomes through meta-analyses have aimed to obtain a more robust conclusion on the role of NAT in R-PDAC. However, results have been inconclusive, and the uncertainty surrounding the role of NAT in R-PDAC persists^7,8^. Published meta-analyses may be influenced by various factors, such as the method of calculating the effect size, timing of follow-up and limited amount of information, potentially leading to unreliable data and an overestimation of the effect size^9^. In these cases, patient-level data meta-analyses are preferred over aggregated data meta-analyses.

The aim of the current meta-analysis was to assess the difference on overall survival (OS) between NAT and US in patients with R-PDAC.

## Methods

### Search strategy and study selection

This review was prospectively registered in PROSPERO (international prospective register of systematic reviews) (CRD42023451092) and conducted in accordance with the Preferred Reporting Items for Systematic Reviews and Meta-Analyses (PRISMA) guidelines^10^ and Cochrane recommendations^11^. A systematic literature review was performed using PubMed, MEDLINE (Ovid) and Web of Science to retrieve studies published from database inception up to 1 August 2023, following a rigorous search strategy (see *Appendix*). Relevant reviews and studies were manually cross-referenced to identify any additional eligible studies, which included RCTs comparing OS between patients diagnosed with R-PDAC depending on whether they received NAT or US. For study inclusion, studies had to be in English, published in peer-reviewed journals, and include at least one group of patients receiving neoadjuvant CMT or chemoradiotherapy and another group undergoing US. All studies had to include patients with histologically or cytologically proven pancreatic cancer at randomization, and the resectability criteria should have been determined by high-quality imaging tests (contrast-enhanced computed tomography scans). Studies also had to provide intention-to-treat (ITT) survival information and report data using Kaplan–Meier curves with details on the number of patients at risk, survival rates, and either the log-rank test or hazard ratio (HR) statistics. The study selection process is detailed in the *Supplementary material*.

### Risk-of-bias assessment

To assess the quality of the selected studies, Version 2 of the Cochrane risk-of-bias tool for RCTs (RoB 2) was utilized^12^. Evaluation was independently conducted by two reviewers (D.A. and N.B.) in duplicate, and disagreements were resolved through consensus. To visually represent the risk-of-bias assessments, ‘traffic light’ plots were generated using the robvis tool^13^.

### Extraction and reconstruction of individual participant data

Reconstruction of time-to-event observations was performed based on data extracted from published Kaplan–Meier plots. Survival data was extracted from these plots using DigitizeIt® software. To reconstruct the survival data an iterative algorithm as described by Guyot *et al*. was employed. By utilizing this algorithm, patient-level survival data were transformed to estimate time-to-event parameters while preserving monotonicity^14–16^ (see *Supplementary material* for details).

### Statistical analysis

Frequentist and Bayesian approaches were used to conduct the survival analysis on an ITT basis. To estimate the time-to-event outcomes, the Kaplan–Meier product-limit model was utilized. To compare the unadjusted OS between groups, the log-rank test was applied. All analyses were carried out using STATA version 16 (StataCorp, College Station, TX, USA).

#### Frequentist analysis

One-stage meta-analysis was performed using both semiparametric and non-parametric models. Hazard ratios were calculated through Cox proportional hazards models, and 95% c.i. were estimated. The primary analysis of OS was based on a γ-shared-frailty Cox regression model. This enables the accommodation of dissimilarities in baseline rates among participants due to unmeasured covariates. Frailty terms maintain the uniform shape of baseline hazards across studies while allowing for varying magnitudes of these hazards^17^. For sensitivity analysis, a stratified model as well as the marginal model were employed. An independent survival analysis was performed to exclude studies with a high risk of bias. To assess the proportionality of hazards assumption, scaled Schoenfeld residuals were visually plotted, and the predicted *versus* observed survival functions were examined. Quantitative assessment was also performed using the Grambsch–Therneau test^18^. To compute the between-group contrast measures from the restricted mean survival time (RMST), the naive Kaplan–Meier method was used as an alternative to HR. The median survival time with a 95% c.i. was also calculated. Two-stage meta-analysis of independent study-based HRs was conducted using a fixed-effects model (inverse variance). To quantify the degree of heterogeneity across studies, the Higgins statistic (*I*^2^) was used. *I*^2^ values of 25%, 50% and 75% represented low, moderate and high heterogeneity respectively^19^. Publication bias was assessed visually using funnel plots and quantitatively using Egger's test. All statistical tests were two-sided, and the significance level was set at 0.05.

#### Bayesian analysis

A fully parametric survival regression model was chosen. The Weibull distribution was selected due to its ability to combine both the accelerated failure time and proportional hazard properties, offering the advantage of deducing HRs using Cox regression while also accounting for accelerated failure time. A marginal and shared frailty model was utilized. Bayesian analysis was conducted with two chains, using 52 500 Monte Carlo Markov Chain (MCMC) iterations after a 50 000 MCMC simulation run (see *Supplementary material* for details).

## Results

### Systematic search and study characteristics

**Fig. 1:**
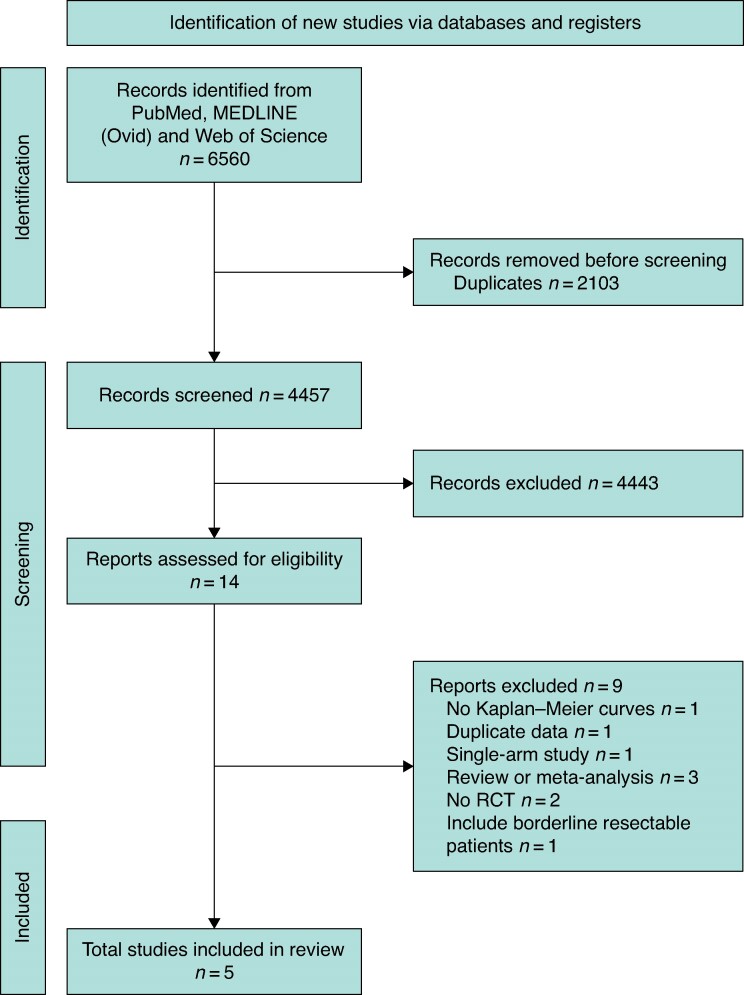
PRISMA flow chart according to guidelines RCT, randomized clinial trial.

The initial search yielded 6560 articles. After removing duplicates, 4457 articles remained. Five RCTs including 625 patients met all inclusion criteria and were included in the final analysis^4,6,20–22^ (*Fig. 1*). The excluded studies and reasons for rejection are shown *Table S1*. Three RCTs are categorized as phase II trials^20–22^. One trial is classified as phase II/III^6^ and another is a phase III RCT^4^.

### Patients and treatment characteristics

Of the 625 patients, 272 patients underwent US and 353 received NAT. In the neoadjuvant arm, 288 patients received CMT alone, while 65 received a combination of CMT and radiation therapy (RDT)^4^. Among patients treated with CMT, 147 received the FOLFIRINOX regimen, 50 were treated with FOLFOX, and the remaining 156 patients received various CMT protocols primarily based on gemcitabine. *Table 1* shows the detailed patient and study information.

**Table 1:** Patient and study characteristics

Study (year)	Resectability criteria	Intervention (*n*)	Patient characteristics
Seufferlein *et al.* (2023)^21^	Clear fat planes around the celiac artery, hepatic artery and superior mesenteric artery	2 cycles of nab-paclitaxel/gemcitabine (nab-paclitaxel 125 mg/m^2^, gemcitabine 1000 mg/m^2^ on day 1, 8, and 15 of a 28 day cycle)* (59)	Median CA19-9: 175 U/ml
		Upfront surgery (59)	Median CA19-9: 134 U/ml
Labori *et al.* (2023)^20^	T1-3, Nx, M0 (UICC 7th version, 2010)	4 cycles of FOLFIRINOX (77)	Median age: 66.5 years (i.q.r. 59–72) 115 (82.1%) and 25 (17.9%) patients were ECOG 0 and 1 respectively
		Upfront surgery (63)	
Versteijne *et al.* (2022)^4^	Tumour contact with the superior mesenteric vein or portal vein was £90° without any arterial contact	3 cycles with gemcitabine at a dose of 1000 mg/m^2^. The second cycle with gemcitabine at a dose of 1000 mg/m^2^, combined with hypofractionated radiotherapy (36 Gy in 15 fractions) (65)	Mean age (years): 66 (i.q.r. 59–71) Male *n* (%): 64 (54) Female *n* (%): 55 (46) Resectable *n* (%): 65 (55) Borderline resectable *n* (%): 54 (45) Baseline CA19-9 ^3^500 U/ml *n* (%) 31 (29)
		Upfront surgery (68)	Mean age (years): 67 (i.q.r. 60–73) Male *n* (%): 77 (61) Female *n* (%): 53 (42) Resectable *n* (%) 68 (54) Borderline resectable *n* (%) 59 (47) Baseline CA19-9 ^3^500 U/ml *n* (%) 35 (35)
PANACHE01-PRODIGE48 study (2022)^22^	Absence of distant organ or distal lymph node metastases, absence of evidence of superior mesenteric vein and portal vein distortion, tumour thrombus or venous encasement, the existence of clear fat planes around the celiac axis, hepatic artery and superior mesenteric artery	4 cycles of FOLFOX (50)	NR
		4 cycles of FOLFIRINOX (70)	NR
		Upfront surgery (26)	NR
Reni *et al.* (2018)^6^	Absence of invasion of superior mesenteric artery or vein, portal vein, coeliac artery or hepatic artery	Intravenous cisplatin 30 mg/m^2^, epirubicin 30 mg/m^2^ and gemcitabine 800 mg/m^2^ on days 1 and 15 every 4 weeks, and oral capecitabine 1250 mg/m^2^ on days 1–28. (32)	Mean age (years): 64 (i.q.r. 39–75) Male *n* (%): 25 (78) Female *n* (%): 7 (22) Baseline CA19-9 U/ml: 173 (43–4510)
		Upfront surgery (26)	Mean age (years): 65 (i.q.r. 37–74) Male *n* (%): 14 (54) Female *n* (%): 12 (46) Baseline CA19-9 U/ml: 179 (39–3337)
		Upfront surgery (30)	Mean age (years): 68 (i.q.r. 49–75) Male *n* (%): 13 (43) Female *n* (%): 17 (57) Baseline CA19-9 U/ml: 240 (40–12 000)

Values are *n* (%) unless otherwise indicated. ITT, intention to treat analysis; NAT, neoadjuvant therapy; NR, not reported; i.q.r., interquartile range. *The final analysis was performed on a non-prespecified mITT analysis including patients who had received at least one dose of adjuvant or neoadjuvant therapy.

### Study risk-of-bias assessment

*Table S2* shows the bias risk assessment for each study. Four studies had a low risk of bias and one^21^ had a high risk of bias due to deviations from the intended intervention (see *Supplementary material* for details).

### Survival analysis

**Fig. 2:**
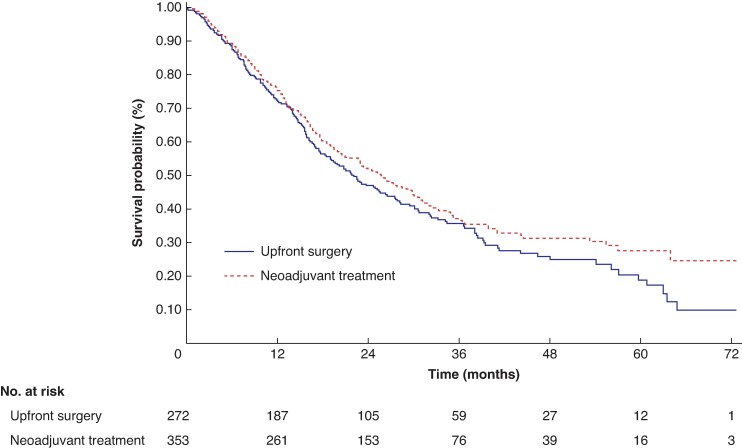
Kaplan–Meier plot and number at risk table for resectable R-PDAC (*n* = 625) *P* = 0.1295. R-PDAC, resectable pancreatic ductal adenocarcinoma.

**Fig. 3:**
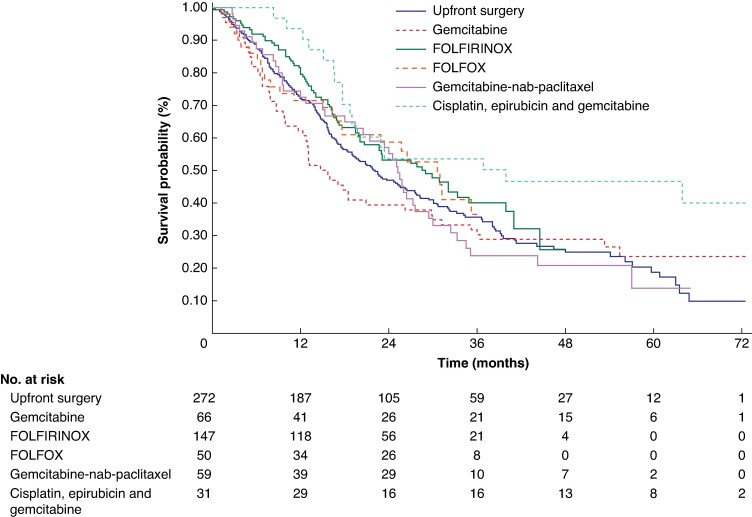
Kaplan–Meier plot and number at risk table for resectable R-PDAC according to NAT protocol (*n* = 625) Only the arm involving cisplatin, epirubicin and gemcitabine showed a statistically significant difference in favour of NAT compared with the upfront surgery arm, with a log-rank *P* value of 0.0059. R-PDAC, resectable pancreatic ductal adenocarcinoma; NAT, neoadjuvant treatment.

Among the 625 patients, the median OS of patients in the NAT and US groups was 25.8 months (95% c.i. 21.4 to 29.9) and 22.1 months (95% c.i. 18.3 to 26.5) respectively. The Kaplan–Meier estimated OS rates at 5 years were 27.6% (95% c.i. 21.2 to 34.4) and 18.8% (95% c.i. 12.3 to 26.4) for the NAT and US groups respectively, with a log-rank *P* value of 0.1295 (*Fig. 2*). Based on the NAT protocols utilized, the analysis revealed that only the arm involving cisplatin, epirubicin and gemcitabine showed a statistically significant difference in favour of NAT compared with the US arm, with a log-rank *P* value of 0.0059 (*Fig. 3*).

**Fig. 4:**
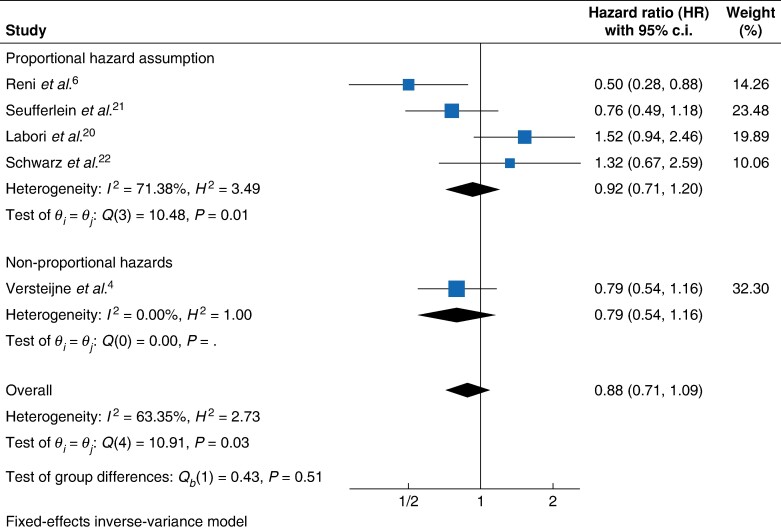
Forest plot of resectable PDAC patients hazard of death fixed-effect meta-analysis PDAC, pancreatic ductal adenocarcinoma.

Shared frailty Cox regression analysis revealed no statistically significant difference in the hazard of death provided by NAT (HR 0.881, 95% c.i. 0.718 to 1.080, *P* = 0.2230) compared with US. Analysis of the marginal (HR 0.857, 95% c.i. 0.703 to 1.046, *P* = 0.1313) and stratified (HR 0.883, 95% c.i. 0.718 to 1.087, *P* = 0.2416) models were consistent, showing no reduction in the hazard of death associated with NAT. The non-parametric analysis was also consistent with no significant increases in RMST associated with NAT (+2.41 months, 95% c.i. −1.22 to 6.04, *P* < 0.194). In the two-stage meta-analysis, the pooled HR was 0.88 (95% c.i. 0.71 to 1.09, *P* = 0.51) (*Fig. 4*) (*Table 2*).

**Table 2:** Survival analysis using reconstructed patient-level data survival information

One-stage meta-analysis	Effect size (95% c.i.)		*P*
Shared frailty HR*	0.881 (0.718 to 1.080)		0.2230
Marginal HR	0.858 (0.703 to 1.046)		0.1313
Stratified HR	0.884 (0.719 to 1.087)		0.2416
**Two-stage meta-analysis**			
HR (fixed-effects)	0.88 (0.71 to 1.09)		0.2417
HR (fixed-effects) (excluding non-proportional hazard assumption)	0.92 (0.71 to 1.20)		0.511
**Non-parametric models**			
RMST difference (up to 5 years)	+2.41 months (−1.22 to 6.04)		0.194

Bayesian model	Effect size (95% c.i.)		Max Gelman–Rubin Rc
Posterior HR	0.859 (0.699 to 1.045)		<1.1
Shared frailty posterior HR	0.863 (0.700 to 1.050)		1.221

Kaplan–Meier product-limit model	Neoadjuvant therapy arm	Upfront surgery arm	log-rank test *P* = 0.1295
1-year OS	75.2% (95% c.i. 70.3 to 79.4)	72.1% (95% c.i. 66.3 to 77.2)	
3-year OS	36.7% (95% c.i. 31.0 to 42.3)	35.7% (95% c.i. 29.5 to 41.9)	
5-year OS	27.6% (95% c.i. 21.2 to 34.4)	18.8% (95% c.i. 12.3 to 26.4)	

OS, overall survival; HR, hazard ratio; RMST, restricted median survival time; PDAC, pancreatic ductal adenocarcinoma. *Primary analysis.

### Sensitivity analysis

**Fig. 5:**
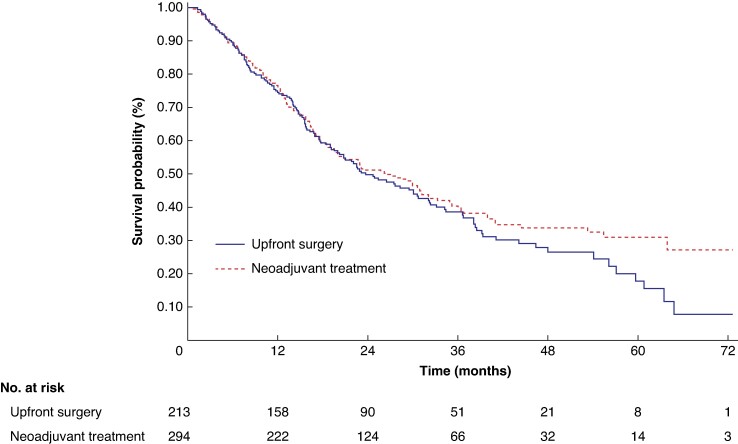
Kaplan–Meier OS curves and number at risk table for resectable PDAC after excluding studies with high risk of bias (*n* = 507) *P* = 0.2638. PDAC, pancreatic ductal adenocarcinoma; OS, overall survival.

After excluding one high-risk study^21^, the analysis focused on 213 and 294 patients in the US and NAT arm respectively. For US patients, the median OS was 23.7 months (95% c.i. 19.6 to 30.7) and the estimated 5-year OS rate was 17.8% (95% c.i. 10.2 to 27.2). For patients in the NAT arm, the median OS was 26.5 months (95% c.i. 20.1 to 31.2) and the estimated 5-year OS rate was 31.1% (95% c.i. 24.1 to 38.3) (log-rank *P* 0.2638; *Fig. 5*). The Cox proportional hazards model revealed no significant reduction in the hazard rate of death associated with NAT (shared frailty HR 0.910 (95% c.i. 0.722 to 1.147; *P* = 0.4242)). The findings were consistent across the rest of the Cox regression and non-parametric survival analyses (*Table S3*).

### Bayesian analysis

The posterior HR for patients with R-PDAC was 0.859 (95% credible interval: 0.699 to 1.045). After conducting Bayesian shared-frailty survival-time regression, the posterior HR remained similar, with a value of 0.863 (95% credible interval: 0.700 to 1.050). Trace plots and the Gelman–Rubin Convergence test confirmed that the convergence criterion was met in the marginal analysis. However, the shared frailty model did not meet the convergence criterion, indicating potential issues with the model's stability (see *Supplementary material* for details).

## Discussion

This meta-analysis, pooling the available highest quality evidence with rigorous methodology, highlights that NAT neither reduces the hazard of death nor results in a statistically significant improvement in long-term OS compared with US in R-PDAC patients.

Upfront surgery followed by adjuvant CMT for 6 months represents the current standard of care treatment for patients with R-PDAC^3,23,24^. However, NAT in R-PDAC patients has been suggested as an alternative to standard treatment, aiming to address subclinical disease and facilitate margin-free resections and allegedly enhancing the delivery of systemic treatment. The rationale underlying the potential benefits of NAT is that PDAC could exhibit substantial systemic involvement even in cases categorized as resectable, a notion supported by the fact that the survival rate of patients with resected PDAC remains low, with a 10-year OS rate of <5%^25^. This strategy aims to enhance survival outcomes and minimize the occurrence of frequent local and metastatic recurrences in R-PDAC^26^. The cumulative advantages of NAT have yielded enhanced survival outcomes in patients with BR-PDAC^27^. Therefore, and despite the distinct biological characteristics, some authors have suggested to extend these prognostic benefits to patients with R-PDAC^28^. However, the advantage of NAT over US among patients with R-PDAC has been controversial and the scientific community has eagerly anticipated the outcomes of well conducted RCTs to establish more robust conclusions.

A compelling argument in favour of NAT has been the administration of treatment before surgery, ensuring a therapy unaffected by complications or early recurrences after surgery. Henry *et al*. highlights that significant postoperative complications following PDAC resection are linked to decreased disease-free interval and OS, with the adverse impact of major complications on survival seemingly attributed to the omission of adjuvant CMT^29^. However, the neoadjuvant approach does not necessarily appear to allow a more effective overall treatment than US and adjuvant CMT, as observed in several of the RCTs included in this meta-analysis in which CMT administration rates between both approaches were not different (*Table S4*)^4,21,22^. Another argument that supporters of NAT defend is its capacity to enhance R0 resection in patients with PDAC. This capacity has been widely investigated in patients with BR-PDAC, in which achieving clear resection margins can be particularly challenging. In this scenario, NAT has been associated with higher rates of achieving R0 resection in patients undergoing surgery, especially when adding RDT^30,31^. Nevertheless, Katz *et al*. demonstrated that this rise in R0 resections associated with RDT did not result in improved survival outcomes^32^. This highlights the fact that systemic control remains the paramount factor for survival in PDAC. Authors that support US in R-PDAC used this argument to suggest that NAT may be used in tumours too extensive for local control to be achieved through surgery and simultaneously address subclinical disease^33^. They argue that in R-PDAC patients, complete tumour resection is typically attainable through surgery, so local control is not usually the primary challenge.

Current guidelines support NAT in patients with R-PDAC exhibiting unfavourable prognostic factors^3^. Various indicators, including elevated CA19-9 levels, large tumour size and lymph node metastasis, may imply aggressive tumour biology or systemic involvement and seem to be associated with worse survival outcomes^34^. In retrospective studies, NAT has showcased its effectiveness in this aspect by improving survival rates, especially among patients identified with elevated CA19-9 levels and other adverse prognostic indicators^35^. In this scenario, some authors argue that NAT could be used as a test of time/chemoresistance to spare patients with early metastatic spread from what would have been a futile intervention. However, other authors support that this approach may not be effective in R-PDAC patients, given the tumour's high rates of chemoresistance and chemotolerance within an immunosuppressive environment and that individualized adjuvant therapy, guided by molecular study of the tumour, may be the most effective option^36^.

The selection of CMT neo/adjuvant regimens is also a topic of current debate. This study is the first to find that NAT with cisplatin, epirubicin and gemcitabine is the only one associated with significant OS superiority compared with US in R-PDAC. Despite the similar inclusion criteria within included RCTs, this result could derive from baseline, stage or tumour differences between NAT subgroup patients, so cautious interpretation is necessary. However, future RCTs are needed to elucidate this aspect.

In summary, this complex scenario inevitably shows the limited understanding of the biology of PDAC and supports the idea that contemporary approaches may play a crucial role in enhancing survival outcomes for these patients. Studying the molecular profile of pancreatic cancer as well as increasing the understanding of how resistant neoplastic cell populations emerge after CMT represent future areas of investigation for developing effective therapies in PDAC^36^. On the other hand, new biomarkers such as circulating tumour DNA (ctDNA) measurement and peripheral KRAS mutations represent promising methods to identify high-risk patients with R-PDAC and guide individualized treatment^37–40^.

### Strengths and limitations

By combining individual patient data across studies, enhanced precision and generalizability are achieved, enabling more accurate treatment effect estimates and in-depth exploration of subgroup interactions. This approach derived robust and reliable conclusions that distinguish this study from previous meta-analyses, which were based on aggregate data^7,8,41^. Despite the accuracy of the reconstruction, the reconstructed data may not exactly match those reported in the RCTs. The exclusive inclusion of RCTs minimizes selection bias and allows pooling the highest quality of evidence. Moreover, and despite controlling for confounding variables, variations in trial designs, endpoints and imbalances in patient phenotypes and neoadjuvant protocols across studies could introduce substantial heterogeneity and skew the results. It is important to note that the five trials were designed as superiority trials rather than non-inferiority trials, making it difficult to compare the equivalence of the interventions.

In conclusion, the available evidence does not support the superiority of NAT in R-PDAC over US followed by adjuvant therapy.

## Supplementary Material

zrae087_Supplementary_Data

## Data Availability

Patient-level deidentified survival data were extracted from articles published in peer-reviewed journals. Therefore, no original data are available. Survival data extracted and used in this study can be requested by contacting the corresponding author.
