# Supplementary material for: Neoadjuvant therapy versus upfront surgery in resectable pancreatic cancer: reconstructed patient-level meta-analysis of randomized clinical trials
Source: BJS Open. 2024 Sep 27;8(5):zrae087. doi: 10.1093/bjsopen/zrae087 (PMC11428068; doi:10.1093/bjsopen/zrae087)
Supplement: zrae087_Supplementary_Data [file zrae087_supplementary_data.docx]

**Neoadjuvant Therapy Versus Upfront Surgery in Resectable Pancreatic Cancer: Reconstructed Patient-Level Meta-Analysis of Randomized Controlled Trials**

Daniel Aliseda MD^1^, Pablo Martí-Cruchaga MD^1,2^, Gabriel Zozaya MD ^1,2^, Nuria Blanco MD ^1,2,^ Mariano Ponz PhD^2,3^, Ana Chopitea PhD^2,3^, Javier Rodríguez PhD^2,3^, Eduardo Castañón PhD^2,3^, Fernando Pardo MD^1,2^, Fernando Rotellar PhD^1,2^

1. HPB and Liver Transplant Unit. Department of General Surgery, Clinica Universidad de Navarra, University of Navarra, Pamplona, Spain
2. Institute of Health Research of Navarra (IdisNA), Pamplona, Spain
3. Department of Oncology, Clinica Universidad de Navarra, University of Navarra, Pamplona, Spain

**Corresponding author**

Daniel Aliseda. HPB and Liver Transplant Unit. Department of General Surgery, Clinica Universidad de Navarra, University of Navarra, Av. Pío XII, 36, 31008, Pamplona, Spain. Phone number: 34 948255400-ext 4711-. e-mail: [dalisedaj@unav.es](file:///C:\Users\jenny\Downloads\dalisedaj@unav.es)

**Disclosures**

The authors declare no conflicts of interest with respect to this manuscript.

**SUPPLEMENTARY MATERIAL**

[SUPPLEMENTARY METHODS 2](#_Toc166743366)

[Patient-level survival reconstruction 2](#_Toc166743367)

[*Labori et al (2023)^1^ 3*](#_Toc166743368)

[*Versteijne et al. (2022)^2^ 5*](#_Toc166743369)

[*Seufferlein et al. (2023)^3^ 9*](#_Toc166743370)

[*Schwarz et al. (2022)^4^ 13*](#_Toc166743371)

[*Reni et al (2018)^5^ 16*](#_Toc166743372)

[Study selection 18](#_Toc166743373)

[Statistical analysis 18](#_Toc166743374)

[SUPPLEMENTARY RESULTS 19](#_Toc166743375)

[Resectable PDAC 19](#_Toc166743376)

[Two-stage meta-analysis 22](#_Toc166743377)

[Bayesian survival analysis 23](#_Toc166743378)

[SUPPLEMENTARY TABLES 25](#_Toc166743379)

[Table 1S. Studies excluded from the study 26](#_Toc166743380)

[Table 2S. Risk of bias assessment for the included studies 27](#_Toc166743381)

[Table 3S. Sensitive survival analysis excluding studies with high risk of bias 28](#_Toc166743382)

[Table 4S. Resection rates and adjuvant treatment details 29](#_Toc166743383)

[SUPPLEMENTARY APPENDIXES 30](#_Toc166743384)

[Search strategy 30](#_Toc166743385)

[PROSPERO registered protocol 31](#_Toc166743386)

[PRISMA Checklist 42](#_Toc166743387)

[REFERENCES 45](#_Toc166743388)

# SUPPLEMENTARY METHODS

## Patient-level survival reconstruction

### Labori et al (2023)^1^

(Supplementary Material 1)

**
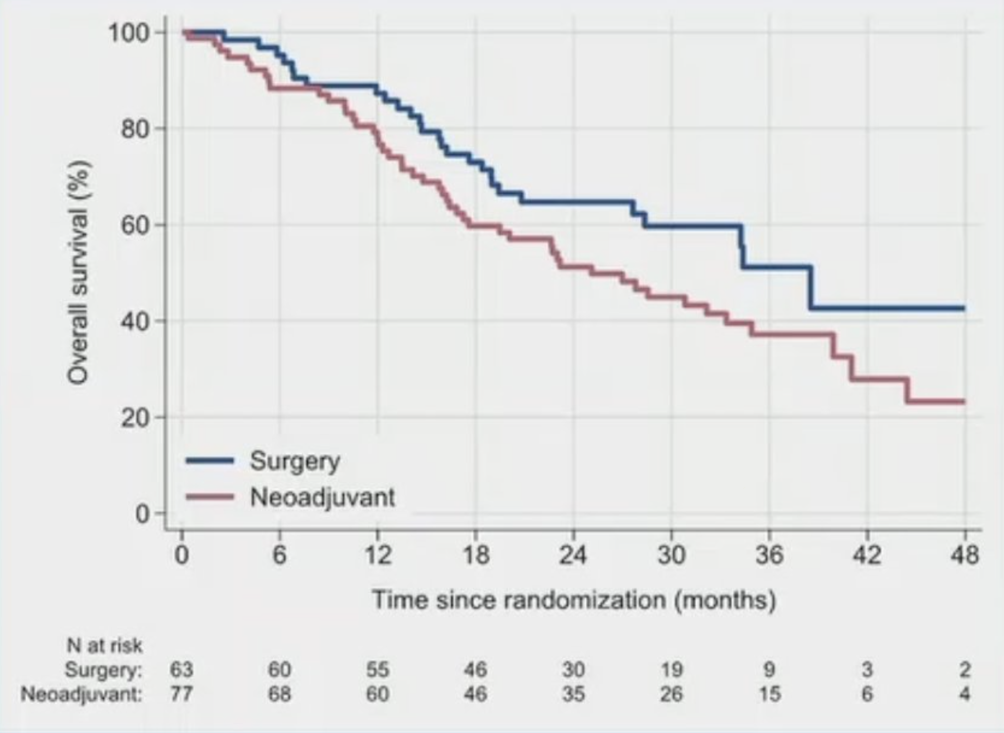
**S1.1. Kaplan–Meier curve from original paper

**
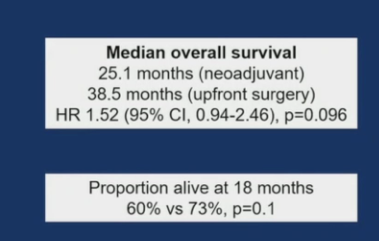
**


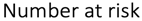

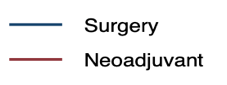
****S1.2. Reconstructed survival curves, including number-at-risk tables

S1.3 Grambsch–Therneau test

**
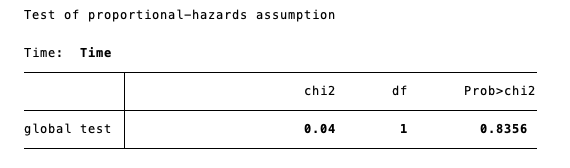
**

S1.4. Schoenfeld residuals plot

S1.5. Predicted versus observed survivor functions

S1.6. Median OS based on reconstructed survival data

**
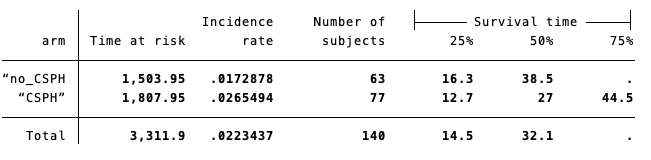
**


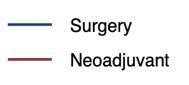


S1.7. Cox regression

**
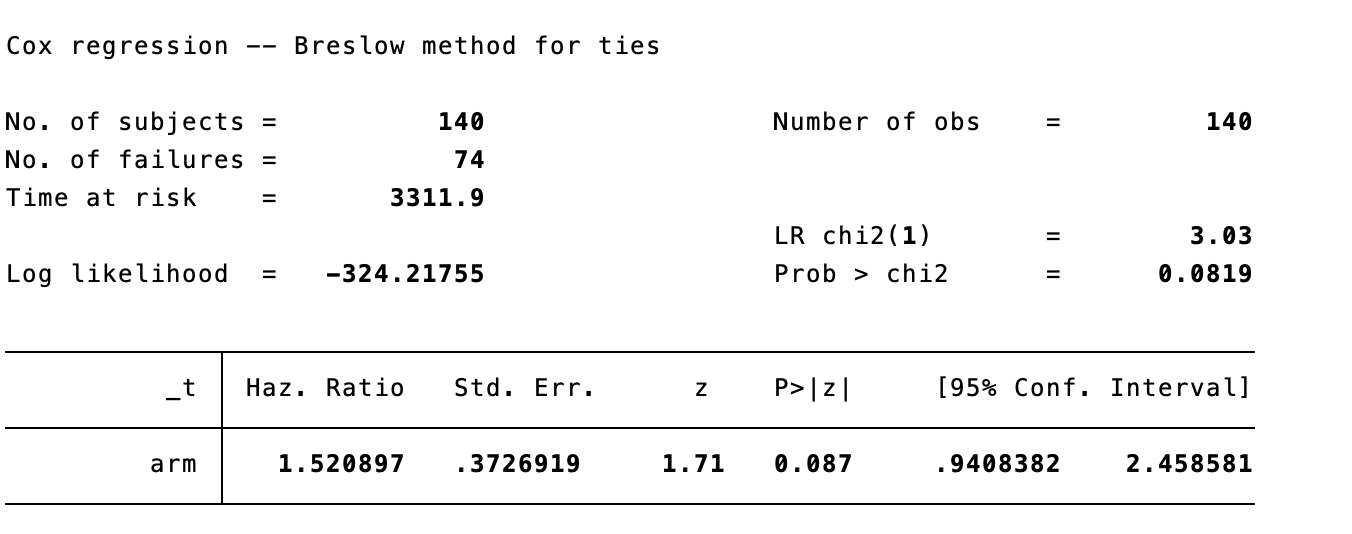
**

### Versteijne et al. (2022)^2^

(Supplementary Material 2)

S2.1. Kaplan–Meier curve from original paper


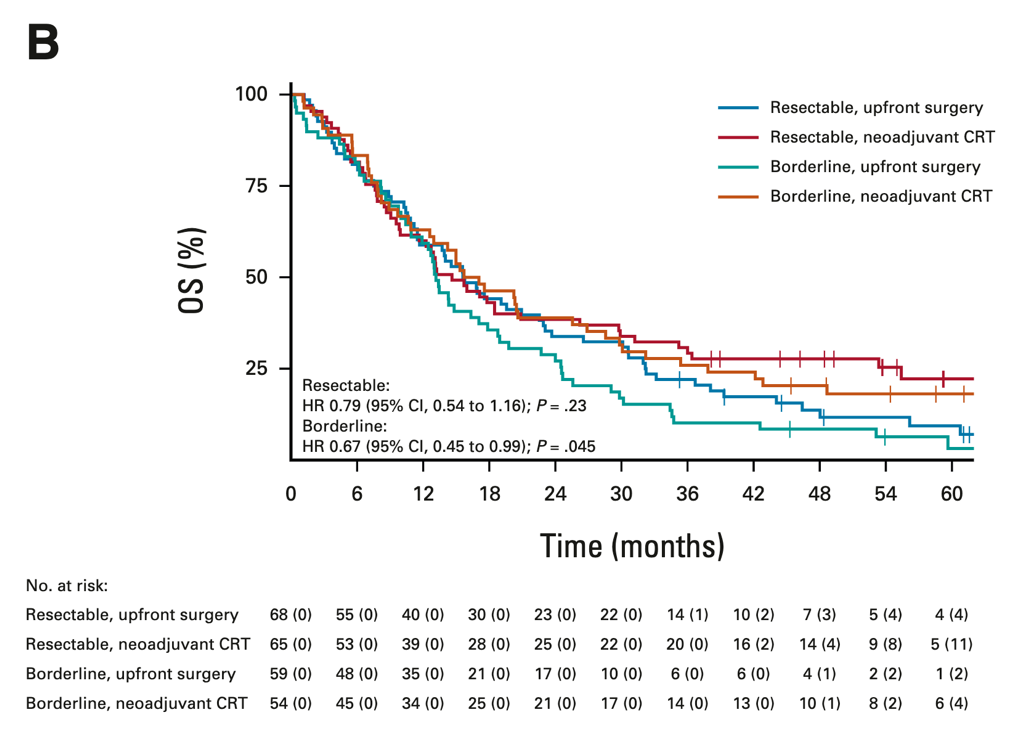


S2.2. Reconstructed survival curves, including number-at-risk tables

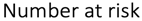


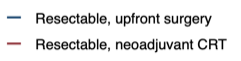


S2.3. Grambsch–Therneau test


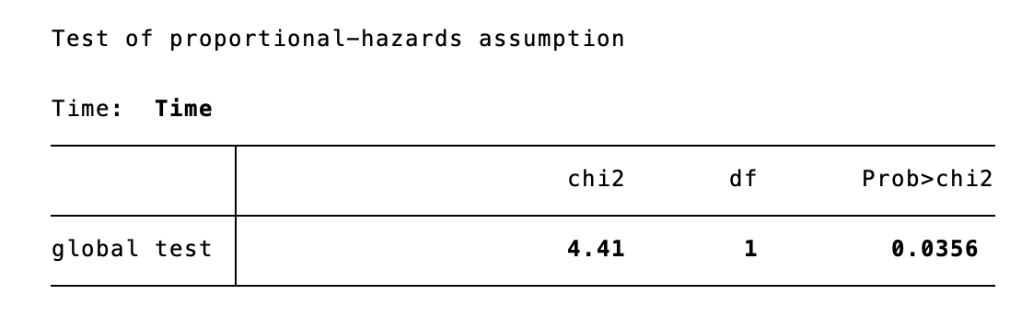


S2.4. Schoenfeld residuals plot

S2.5. Predicted versus observed survivor functions

S2.6. Cox regression


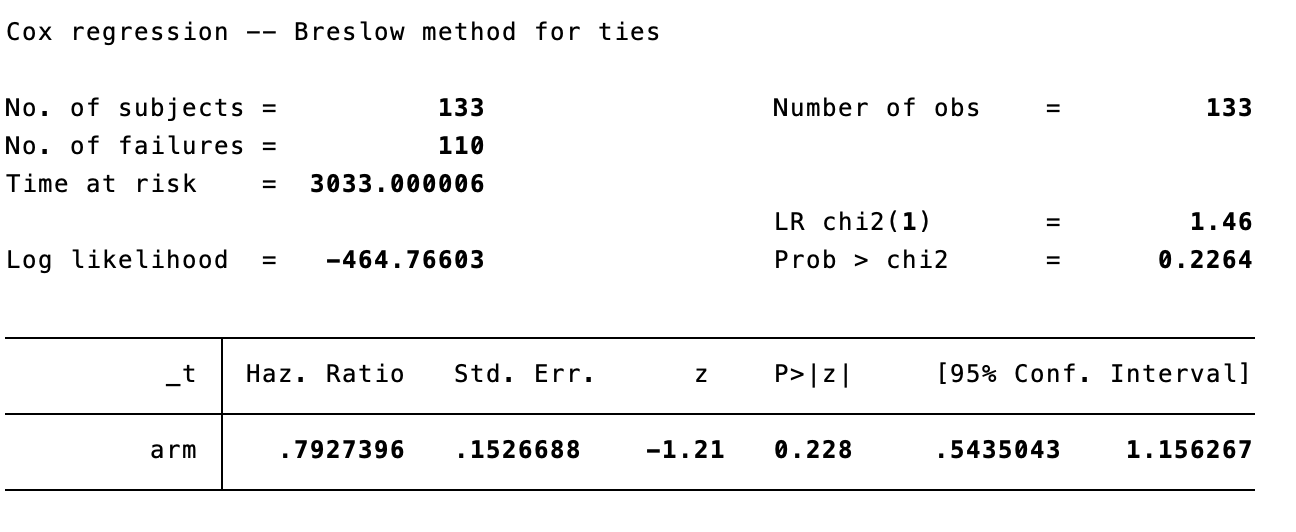


### Seufferlein et al. (2023)^3^

(Supplementary Material 3)

S3.1. Kaplan–Meier curve from original paper -neoadjuvant arm-

**
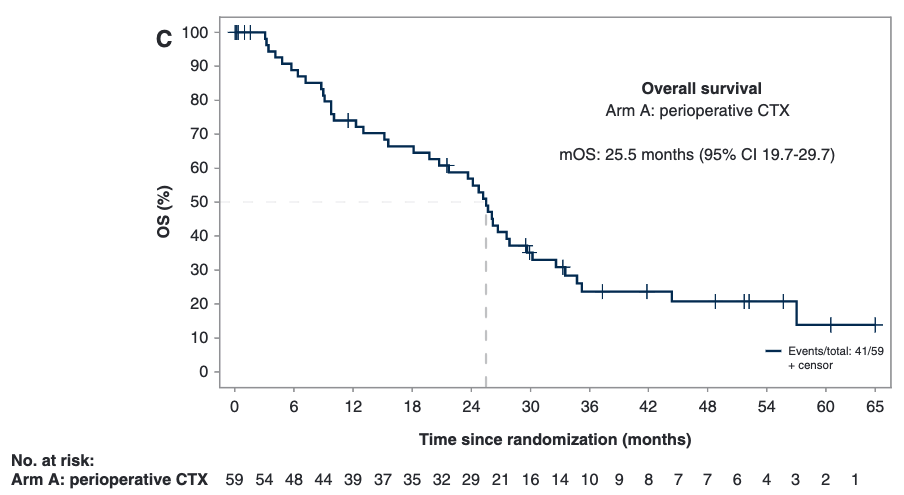
**

S3.2. Reconstructed survival curves, including number-at-risk tables (neoadjuvant arm)

S3.3 Kaplan–Meier curve from original paper -upfront surgery-

**
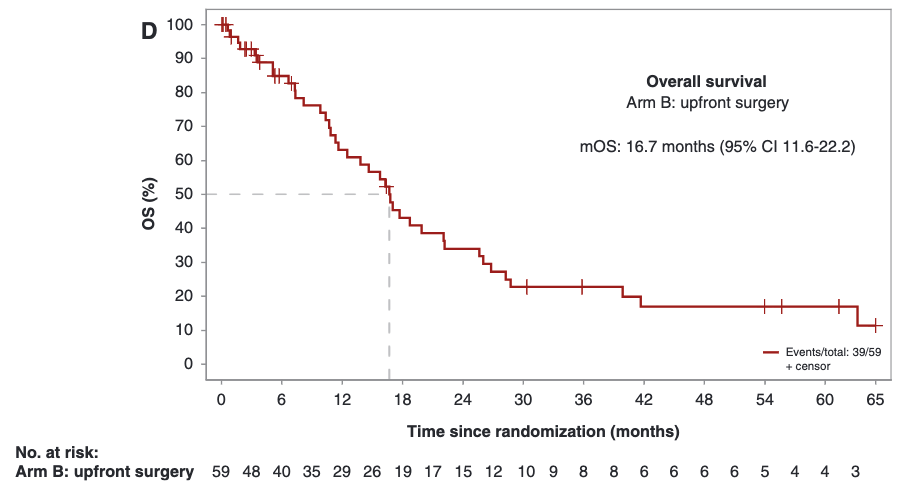
**

S3.4 Reconstructed survival curves, including number-at-risk tables (neoadjuvant)

S3.5 Grambsch–Therneau test

**
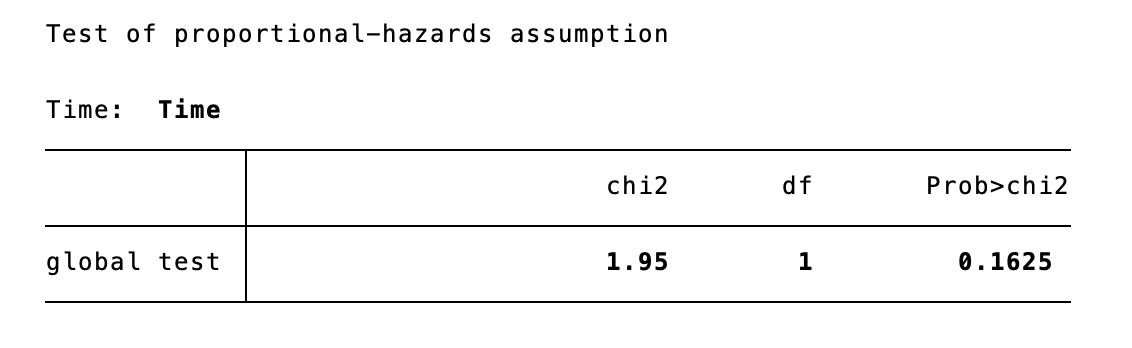
**

S3.6. Schoenfeld residuals plot

S3.7. Predicted versus observed survivor functions

S3.8. Median OS based on reconstructed survival data

**
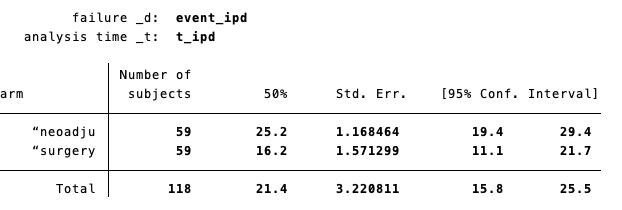
**

S3.9. Cox regression

**
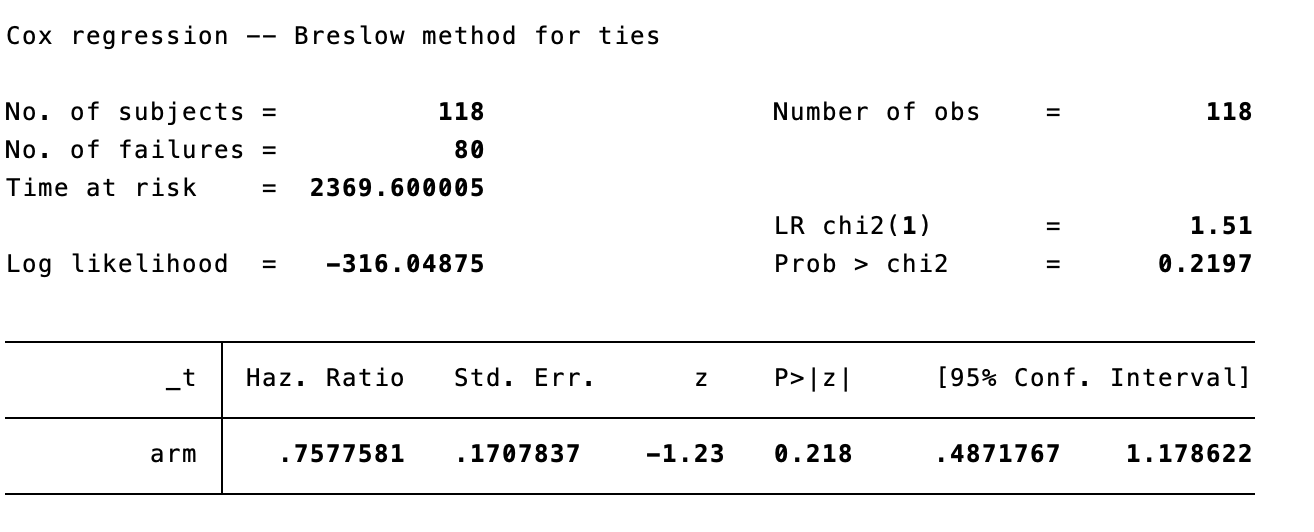
**

### Schwarz et al. (2022)^4^

(Supplementary material. 4)

S4.1. Kaplan–Meier curve from original paper


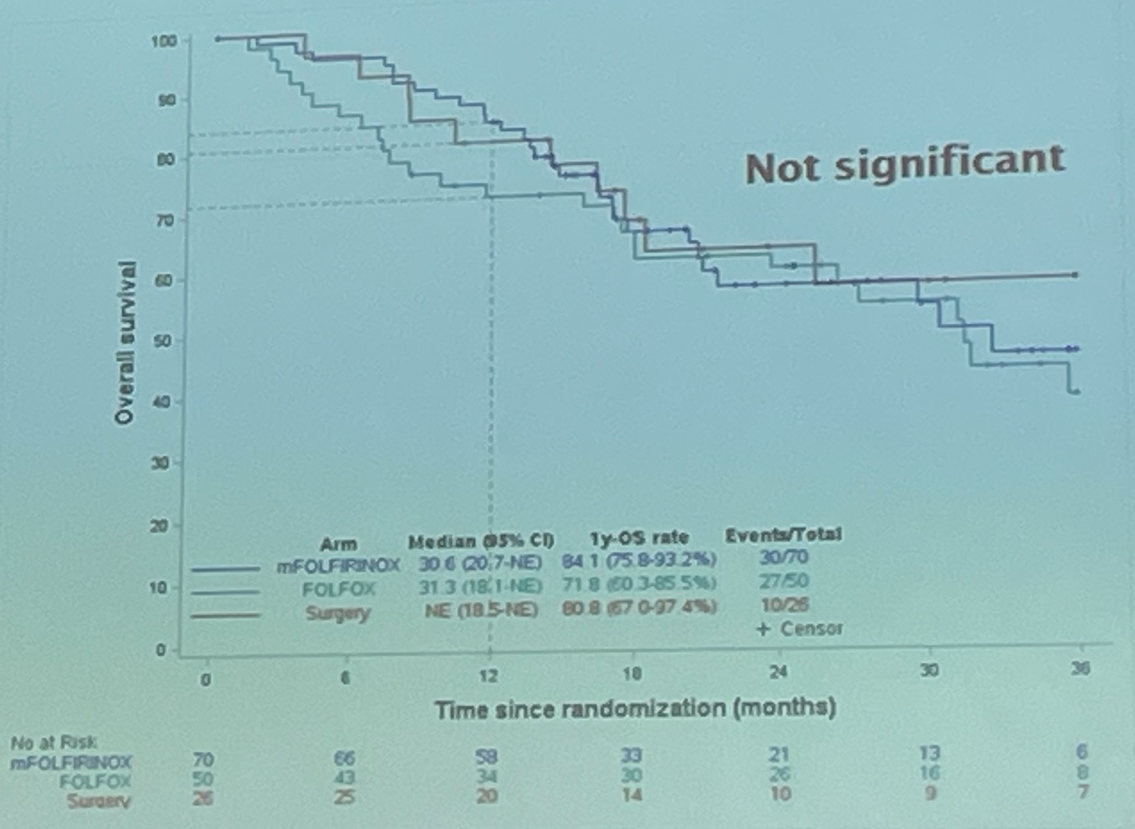


S4.2. Reconstructed survival curves, including number-at-risk tables

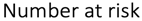


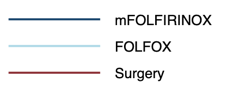


S4.3. Grambsch–Therneau test


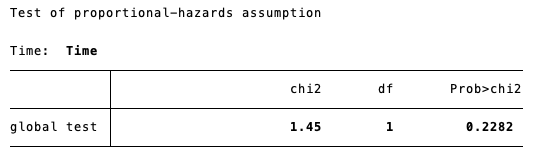


S4.4. Schoenfeld residuals plot

S4.5. Predicted versus observed survivor functions

S4.6. Reconstructed information on Events

**mFOLFIRINOX:** 30/70

**FOLFOX:** 26/50

**Surgery:** 10/26

S4.7. Reconstructed 1-y OS

**1-y OS mFOLFIRINOX:** 82.7% (95% CI: 71.5–89.8)

**1-y OS FOLFOX**: 71.6% (95% CI: 56.6–82.0)

**1-y OS Surgery:** 80.8% (95% CI: 59.8–91.5)

S4.8. Cox regression


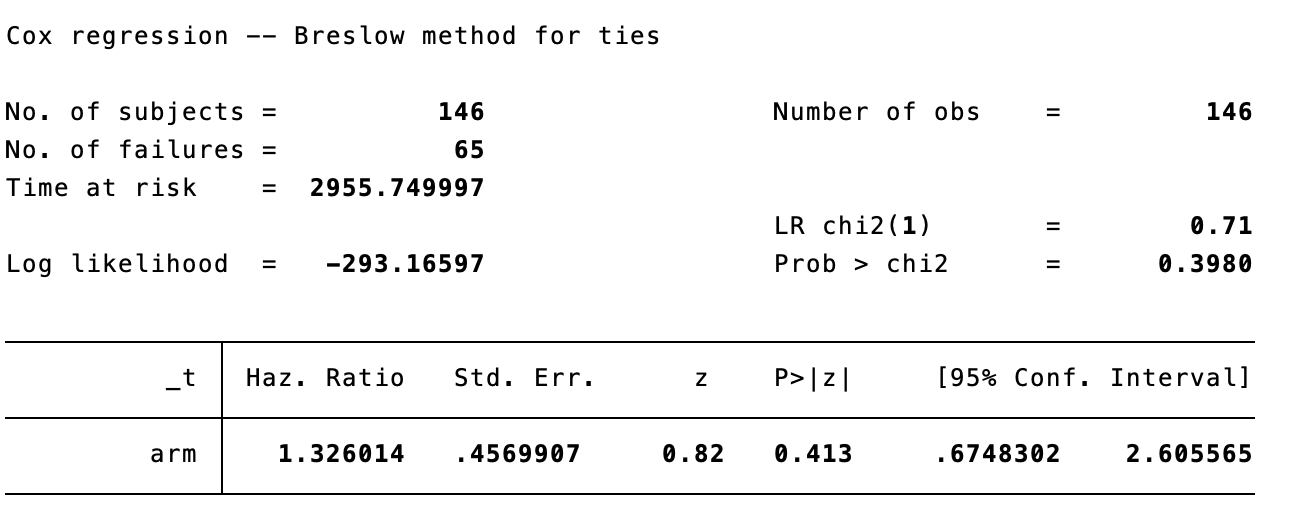


### Reni et al (2018)^5^

(Supplementary Material 5)

S5.1. Kaplan–Meier curve from original paper


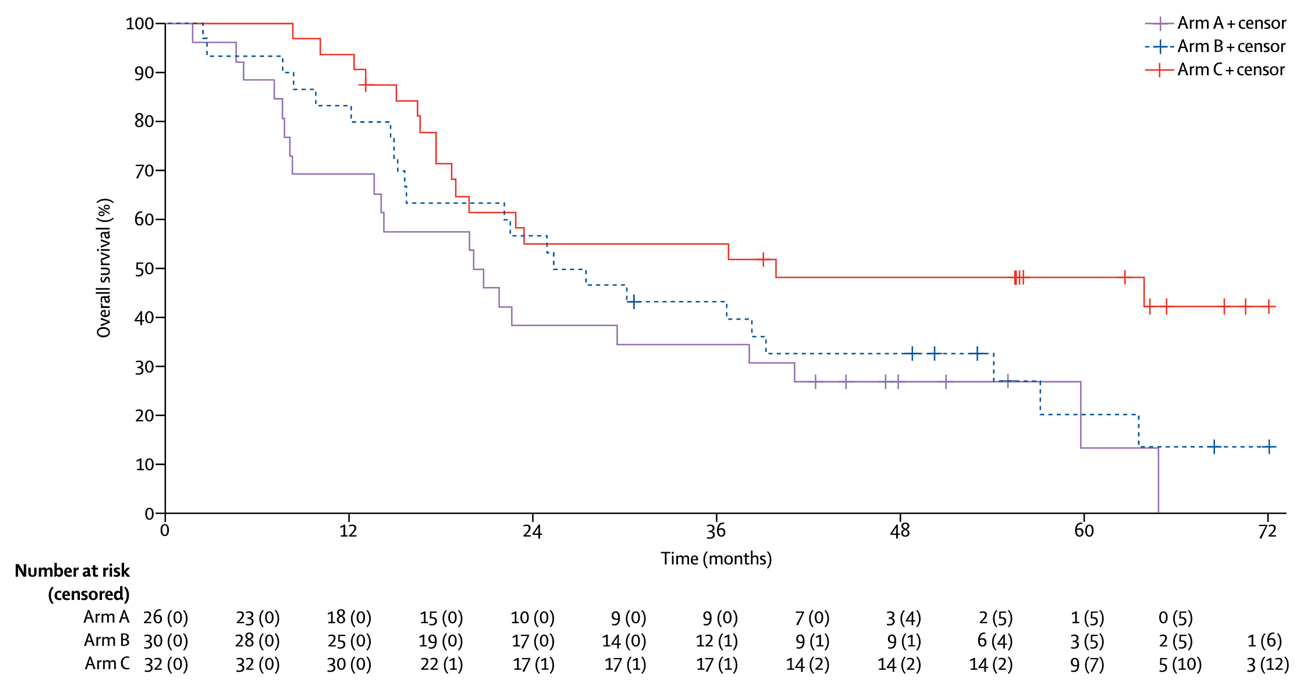


S5.2. Reconstructed survival curves, including number-at-risk tables


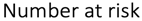

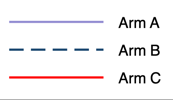


S5.3. Grambsch–Therneau test


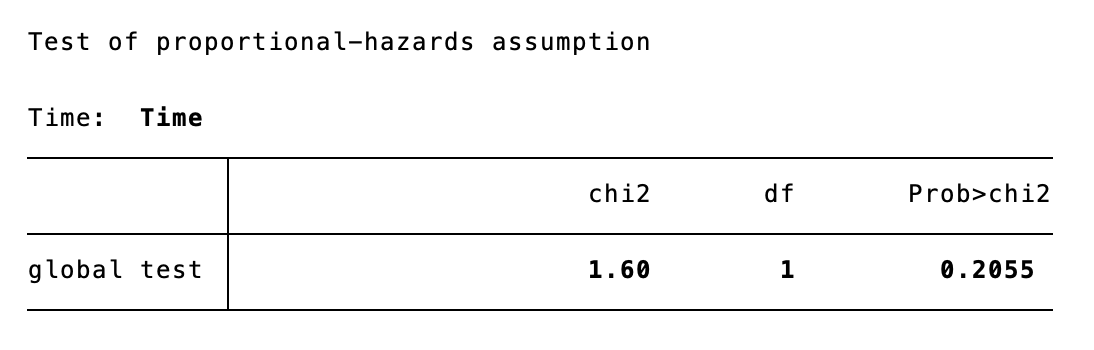


S5.4. Schoenfeld residuals plot

S5.5. Predicted versus observed survivor functions

S5.6. Median OS based on reconstructed survival data


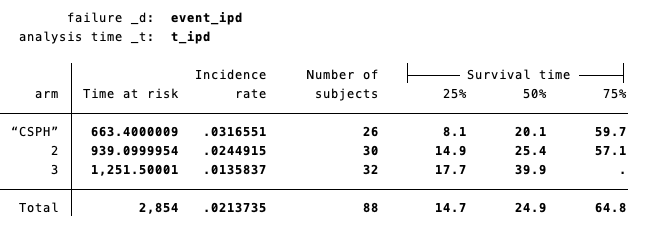


### Study selection

Following an initial double-blind selection process based on titles and abstracts, two reviewers (D.A and N.B) independently applied the inclusion criteria to identify eligible articles. Both reviewers then read the full text of the selected articles, documented reasons for rejecting any remaining articles (Table 1S), and extracted relevant data using a custom form exclusively designed for this review. The extracted data included patient demographics, tumour characteristics (including the resectability criteria in each study), perioperative data, and neo/adjuvant regimen employed.

### Statistical analysis

1. The *ipdfc* command adjusts the values of monotonicity violators using isotonic regression with the pool-adjacent-violators algorithms and replaces adjacent violators with their mean, ensuring that reconstructed data adhere to the monotonicity constraint. Additionally, the command allows for an alternative approach by replacing the value of an offender with the value of its adjacent offender to ensure monotonicity.

2. Bayesian analysis: A normal distribution based on the central limit theorem was used. A noninformative prior was employed, assuming no reduction in the hazard rate and no difference in effect size. This sceptic non-informative prior centered on null effect (HR= 1.00) aimed to avoid overestimating the potential survival benefit. Random-walk Metropolis–Hastings sampling was used, with a 2,500-iteration burn-in to minimize the impact of initial values. A highest density interval (HDI) of posterior distributions with 95% credible intervals was applied. To assess the convergence of the Bayesian analysis, Gelman–Rubin statistics and trace plots were employed. These tools ensure that the MCMC chains have adequately explored the parameter space and reached convergence, validating the reliability of the results^6^

# SUPPLEMENTARY RESULTS

## Resectable PDAC

Reconstructed survival curves, including number-at-risk tables

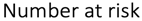


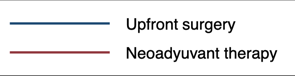


Grambsch–Therneau test


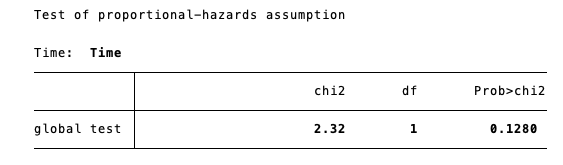


Schoenfeld residuals plot

Predicted versus observed survivor functions

**Sensitivity analysis (excluding high risk of bias studies)**

Reconstructed survival curves, including number-at-risk tables

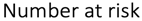


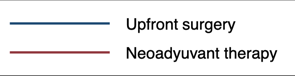


Forest plot of resectable PDAC patients hazard of death fixed effect meta-analysis (excluding high risk of bias studies)

Hazard ratio (HR)

## Two-stage meta-analysis

Forest plot of resectable PDAC patients hazard of death fixed effect meta-analysis

Hazard ratio (HR)

Funnel plot showing publication bias; Hazard ratio (X-axis) with it is standard error (Y-axis)

Egger test

**
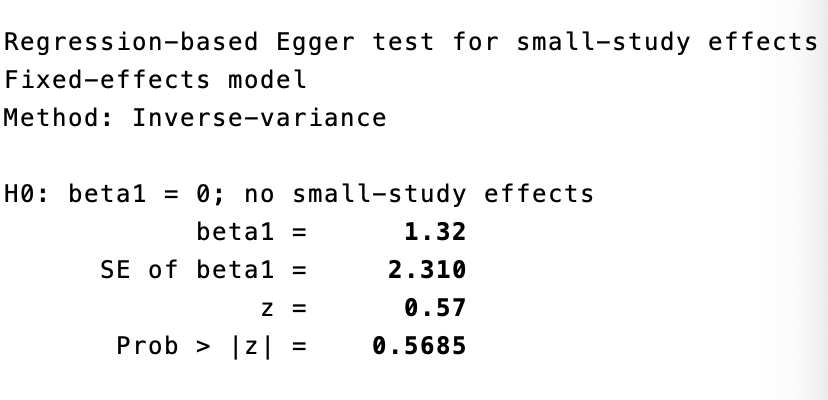
**

## Bayesian survival analysis

Trace, autocorrelation, and distribution plots of Bayesian marginal survival-time regression


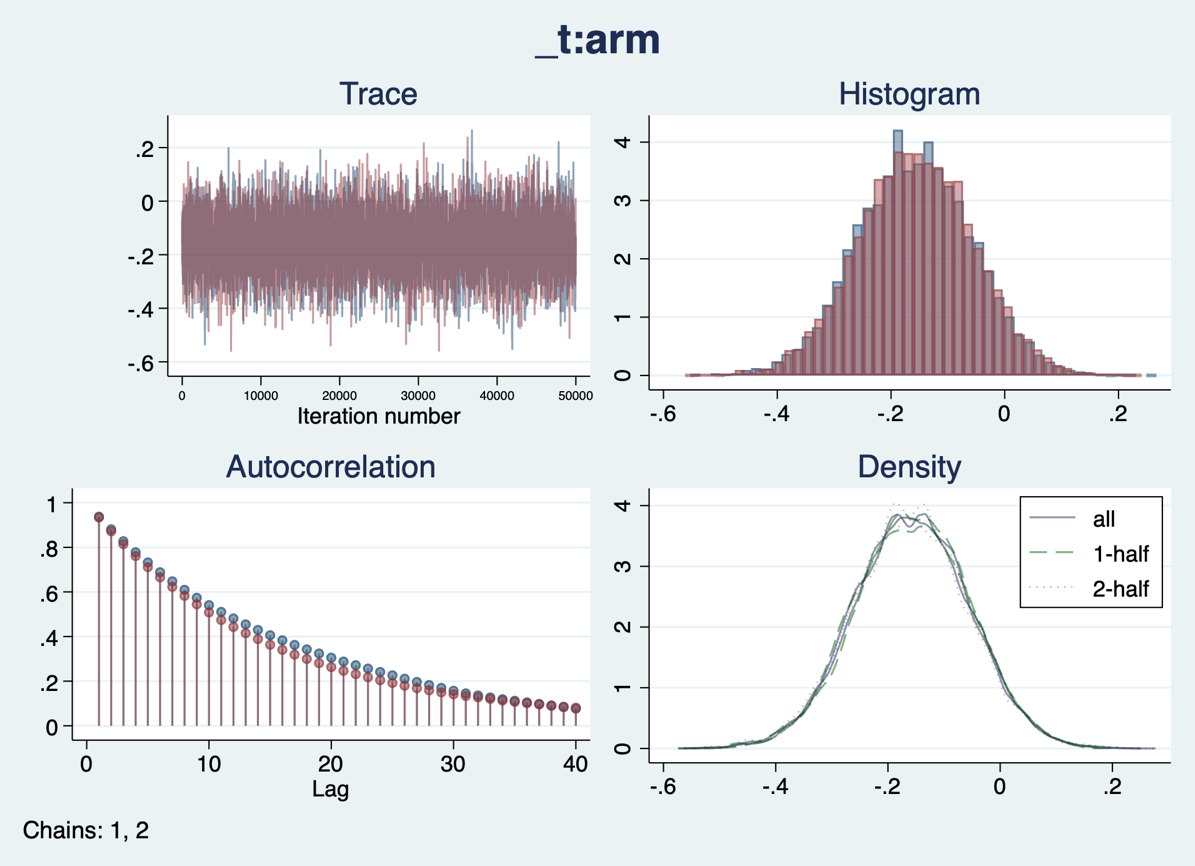


Trace, autocorrelation, and distribution plots of Bayesian shared-frailty survival-time regression


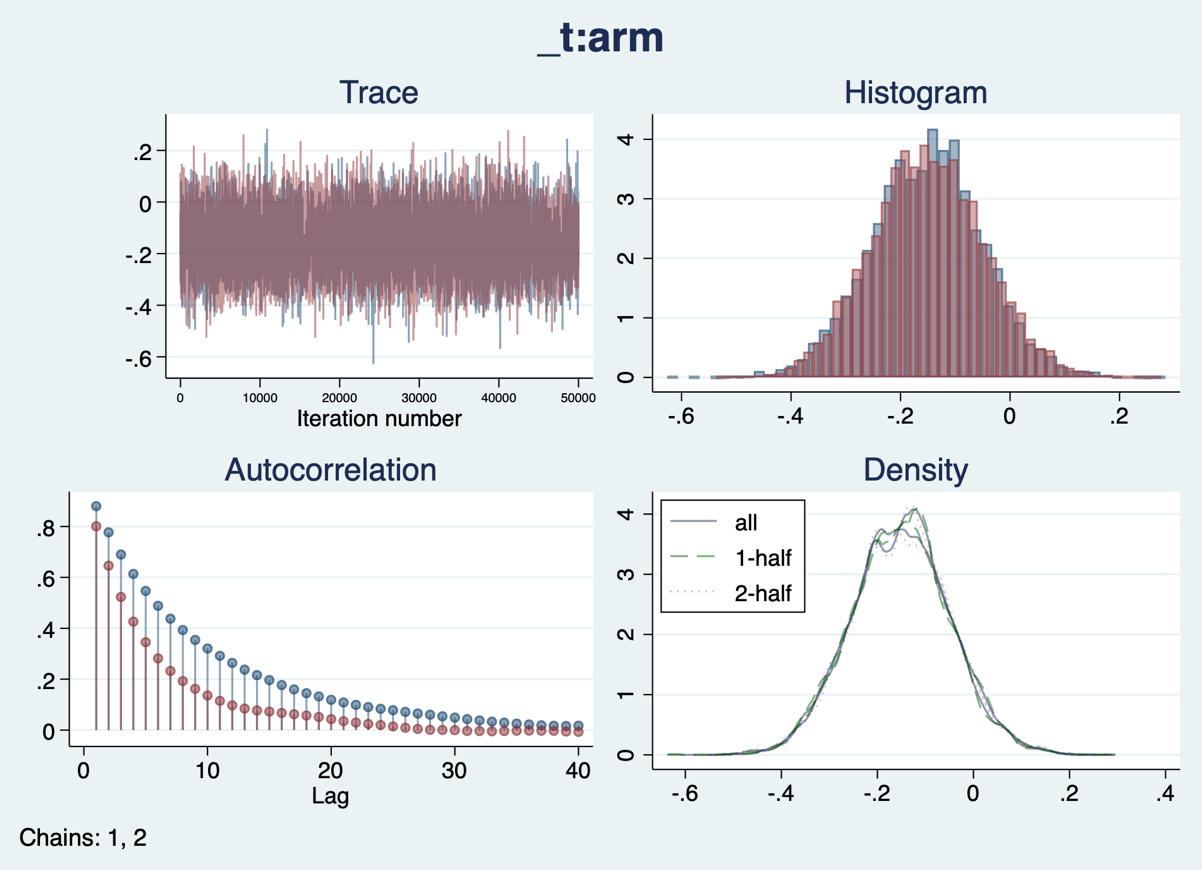


# SUPPLEMENTARY TABLES

| Table 1S. Studies excluded from the study | | |
| --- | --- | --- |
| **Study** | **PMID** | **Cause of exclusion** |
| Neoadjuvant therapy or upfront surgery for resectable and borderline resectable pancreatic cancer: A meta-analysis of randomised controlled trials | **34838371** | Review or meta-analysis |
| Added Value of Radiotherapy Following Neoadjuvant FOLFIRINOX for Resectable and Borderline Resectable Pancreatic Cancer: A Systematic Review and Meta-Analysis | **34142290** | Review or meta-analysis |
| Does neoadjuvant treatment in resectable pancreatic cancer improve overall survival? A systematic review and meta-analysis of randomized controlled trials | **36638709** | Review or meta-analysis |
| Neoadjuvant Chemotherapy versus Upfront Surgery for Resectable Pancreatic Adenocarcinoma: An Updated Nationwide Study | **37226812** | No RCT |
| Neoadjuvant Therapy Followed by Resection Versus Upfront Resection for Resectable Pancreatic Cancer: A Propensity Score Matched Analysis | **27621388** | No RCT |
| Neoadjuvant Chemoradiotherapy and Surgery Versus Surgery Alone in Resectable Pancreatic Cancer: A Single-Center Prospective, Randomized, Controlled Trial Which Failed to Achieve Accrual Targets | **26224039** | No Kaplan–Meier curves |
| Preoperative Chemoradiotherapy Versus Immediate Surgery for Resectable and Borderline Resectable Pancreatic Cancer: Results of the Dutch Randomized Phase III PREOPANC Trial | **32105518** | Duplicate data |
| Results of a Phase II Study on the Use of Neoadjuvant Chemotherapy (FOLFIRINOX or GEM/nab-PTX) for Borderline-resectable Pancreatic Cancer (NUPAT-01) . | **35258510** | Single arm study |
| Oncological Benefits of Neoadjuvant Chemoradiation With Gemcitabine Versus Upfront Surgery in Patients With Borderline Resectable Pancreatic Cancer | **29462005** | Borderline resectable patients |

## Table 2S. Risk of bias assessment for the included studies


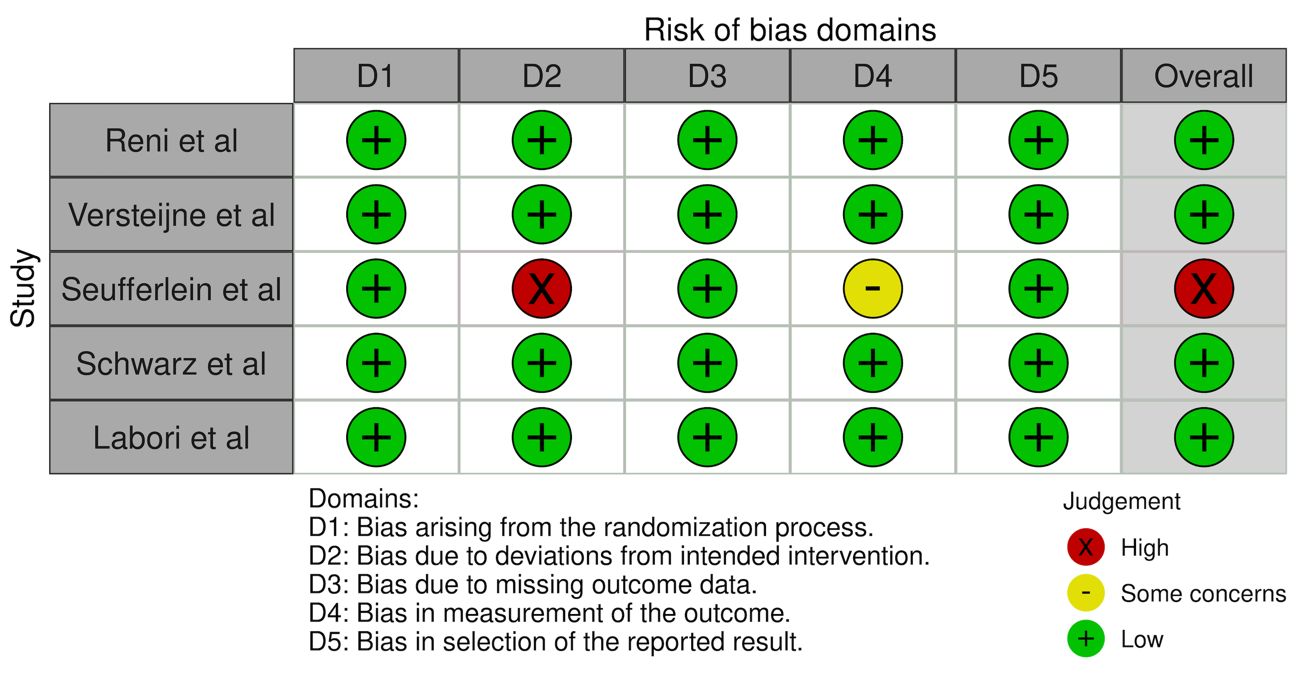


**Reasons to consider high risk of bias**

The NEONAX study by Seufferlein et al. encountered recruitment problems during its course, leading to two amendments that changed the statistical power assumption and sample sizes. In the final analysis, a non-prespecified modified intention-to-treat (mITT) analysis was performed that included patients who had received at least one dose of adjuvant or neoadjuvant therapy. This deviation from the original protocol represents a major protocol violation. According to the NEONAX protocol, patients who did not meet the selection criteria of the trial, including those with local irresectability or distant metastases identified during the study, were supposed to be excluded from the statistical analysis on efficacy. The remaining eligible patients would be evaluated in an intent-to-treat analysis (ITT). However, in the final publication, all patients randomized to the study were included in the ITT analysis, regardless of whether they met the selection criteria or not. This practice introduces a substantial bias into the results.

By including patients who were not eligible based on the protocol, the ITT analysis failed to adhere to the original study design and undermines the validity of the study’s conclusions. The deviation from the prespecified protocol can lead to an overestimation of treatment effects and may affect the study's overall reliability.

## Table 3S. Sensitive survival analysis excluding studies with high risk of bias

| One stage meta-analysis | Effect size (95% CI) | | P-value |
| --- | --- | --- | --- |
| Shared frailty HR* | 0.910 (0.722–1.147) | | 0.4242 |
| Marginal HR | 0.880 (0.704–1.101) | | 0.2659 |
| Stratified HR | 0.922 (0.730–1.165) | | 0.4983 |
| Two stage meta-analysis |  | |  |
| HR (fixed-effects) | 0.92 (0.72–1.18) | | 0.446 |
| Non-parametric models |  | |  |
| RMST difference (up to 5 year) | + 1.86 months (–2.16–5.96) | | 0.360 |
| Kaplan Meier product-limit model | **NAT arm** | **Upfront surgery arm** | log-rank test  p = 0.2638 |
| 1-year OS | 75.7% [95% CI 70.3–80.2] | 74.7% [95% CI 68.2–80.0] |  |
| 3-year OS | 39.7% [95% CI 33.5–45.9] | 38.6% [95% CI 31.6–45.6] |  |
| 5-year OS | 31.1% [95% CI 24.0–38.3] | 17.8% [95% CI 10.2–27.2] |  |
| HR, Hazard ratio; RMST, restricted median survival time; NAT, Neoadjuvant therapy; PDAC: pancreatic ductal adenocarcinoma.  * Primary analysis | | | |

## Table 4S. Resection rates and adjuvant treatment details

| **Table 4S.** Resection rates and adjuvant treatment details | | | |
| --- | --- | --- | --- |
| **Study (*y*)** | **Resection rates**  n (%) | **Adjuvant treatment** | **Initation and completion CMT rates**  n (%) |
| **Seufferlein et al (2023)**^3^ | 41 (69.5) | 4 more cycles of nab-paclitaxel/gemcitabine* | NR |
|  | 46 (78) | 6 cycles of gemcitabine plus nab-paclitaxel (gemcitabine 1,000 mg/m^2^, nab-Paclitaxel 125 mg/m^2^ on day 1, 8, and 15 of a 28 day-cycle) starting within 12 weeks after surgery | NR |
| **Labori et al (2023)**^1^ | 63 (81.8) | mFOLFIRINOX (8 cycles). | Initation: 50 (66.2) |
|  | 56 (88.9) | mFOLFIRINOX (12 cycles). | Initation: 47 (74.6) |
| **Versteijne et al (2022)**^2^ | NR | Gemcitabine (in cycles of 4 weeks) with gemcitabine once weekly in the ﬁrst 3 weeks at a dose of 1,000 mg/m^2^ and was to be started. | Initation: 55 (81)  Completion: 34 (32) |
|  | NR | Gemcitabine (in cycles of 4 weeks) with gemcitabine once weekly in the ﬁrst 3 weeks at a dose of 1,000 mg/m^2^ and was to be started. | Initation: 65 (79)  Completion: 35 (54) |
| **PANACHE01-PRODIGE48 study (2022)**^4^ | 34 (68) | 8 cycles of chemotherapy | Initation: 45 (91) |
|  | 52 (74) | 8 cycles of chemotherapy. | Initation: 62 (88.4) |
|  | 21 (81) | 8 cycles of chemotherapy | Initation: 22 (85.7) |
| **Reni et al (2018)**^5^ | 27 (84) | Intravenous cisplatin 30 mg/m^2^, epirubicin 30 mg/m^2^ and gemcitabine 800 mg/m^2^ on days 1 and 15 every 4 wks, and oral capecitabine 1,250 mg/m^2^ on days 1–28 | NR |
|  | 22 (85) | Gemcitabine 1,000 mg/m^2^ on days 1, 8, and 15 every 4 wks | NR |
|  | 27 (90) | Intravenous cisplatin 30 mg/m^2^, epirubicin 30 mg/m^2^ and gemcitabine 800 mg/m^2^ on days 1 and 15 every 4 wks, and oral capecitabine 1250 mg/m^2^ on days 1–28 | NR |
| * The final analysis was performed on a nonprespecified mITT analysis including patients who had received at least one dose of adjuvant or neoadjuvant therapy.  + Patients that start CMT after surgery. ^Completion of all prespecified adjuvant CMT cycles.  NR: Not reported; DFS: Disease free-survival; OS: Overall survival; CMT: chemotherapy | | | |

# SUPPLEMENTARY APPENDIXES

## **Search strategy**


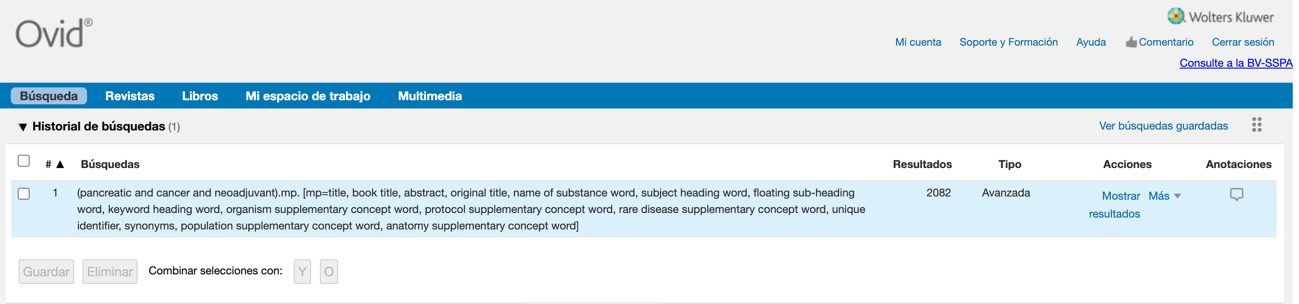


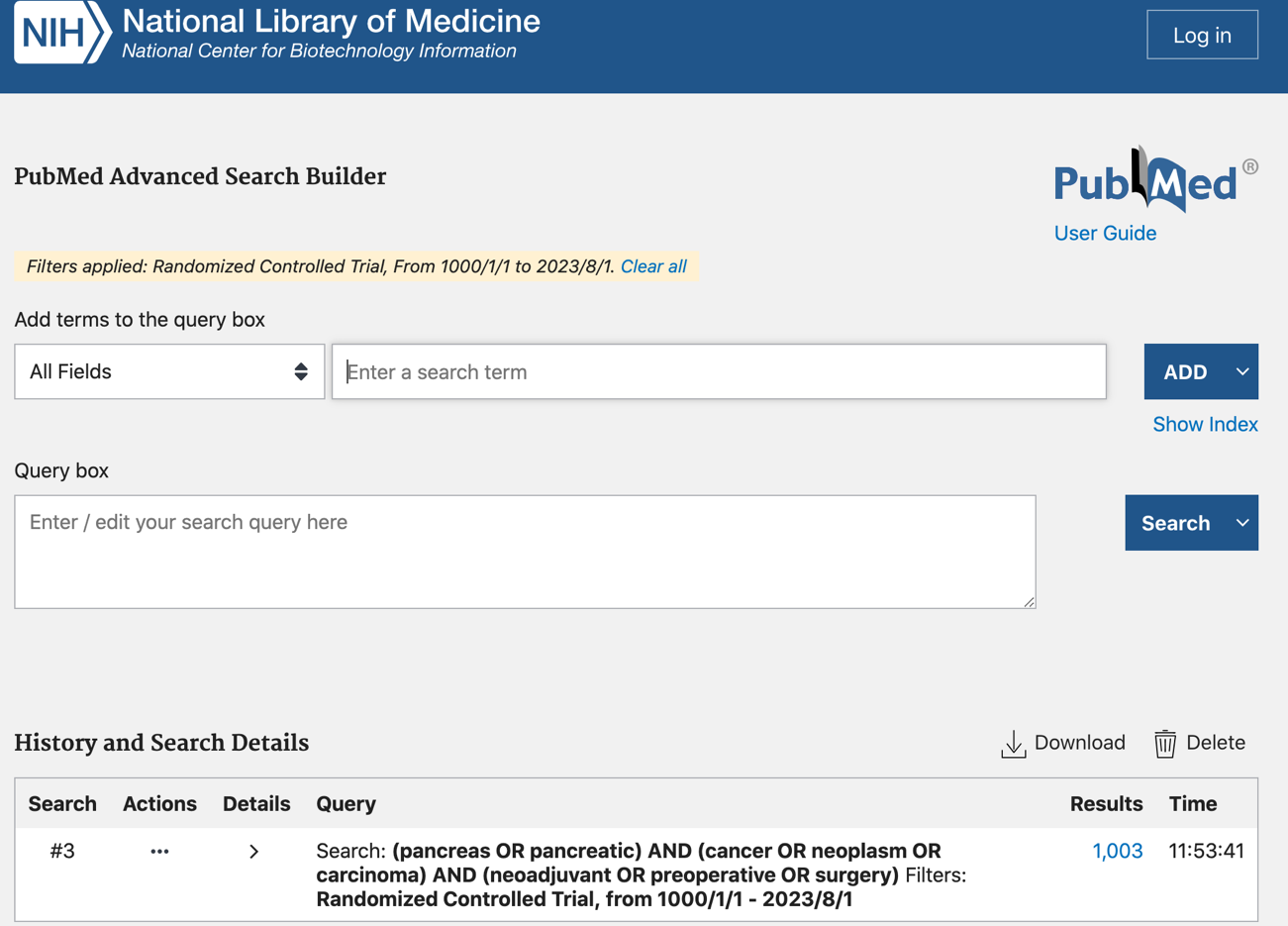


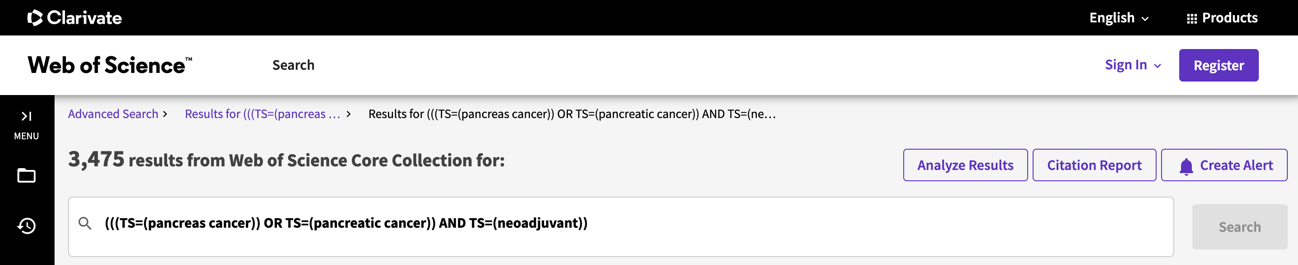


## PROSPERO registered protocol


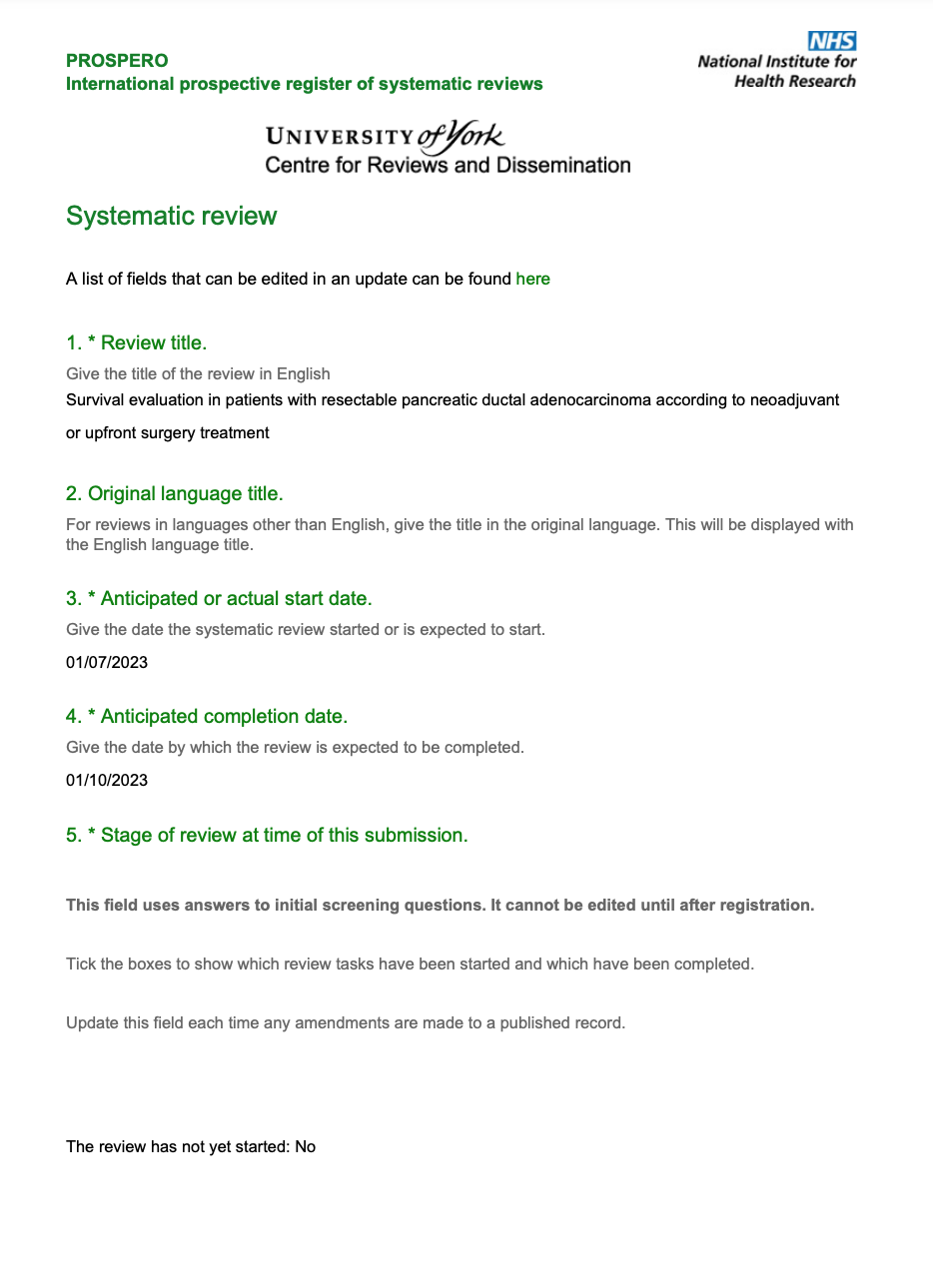


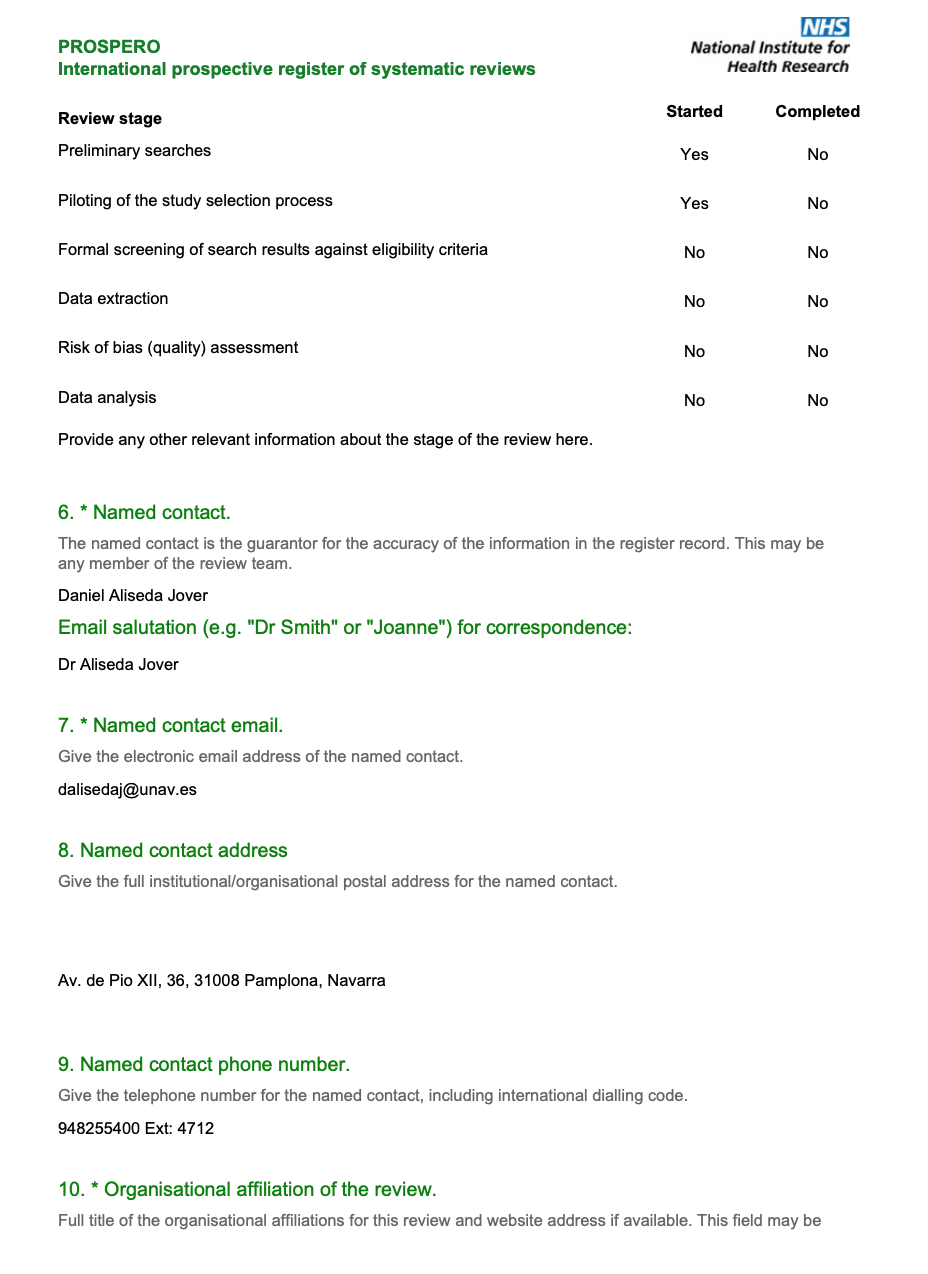


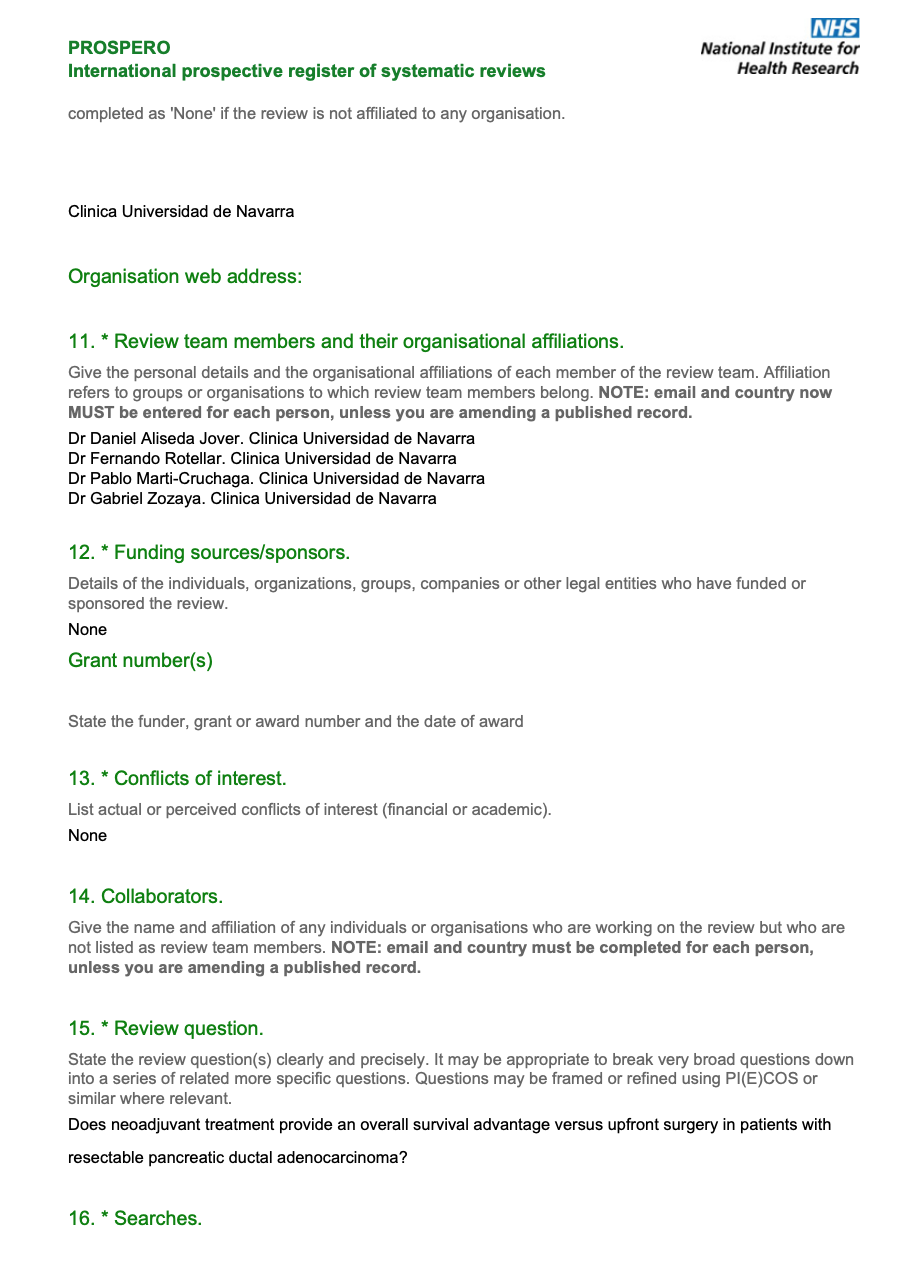


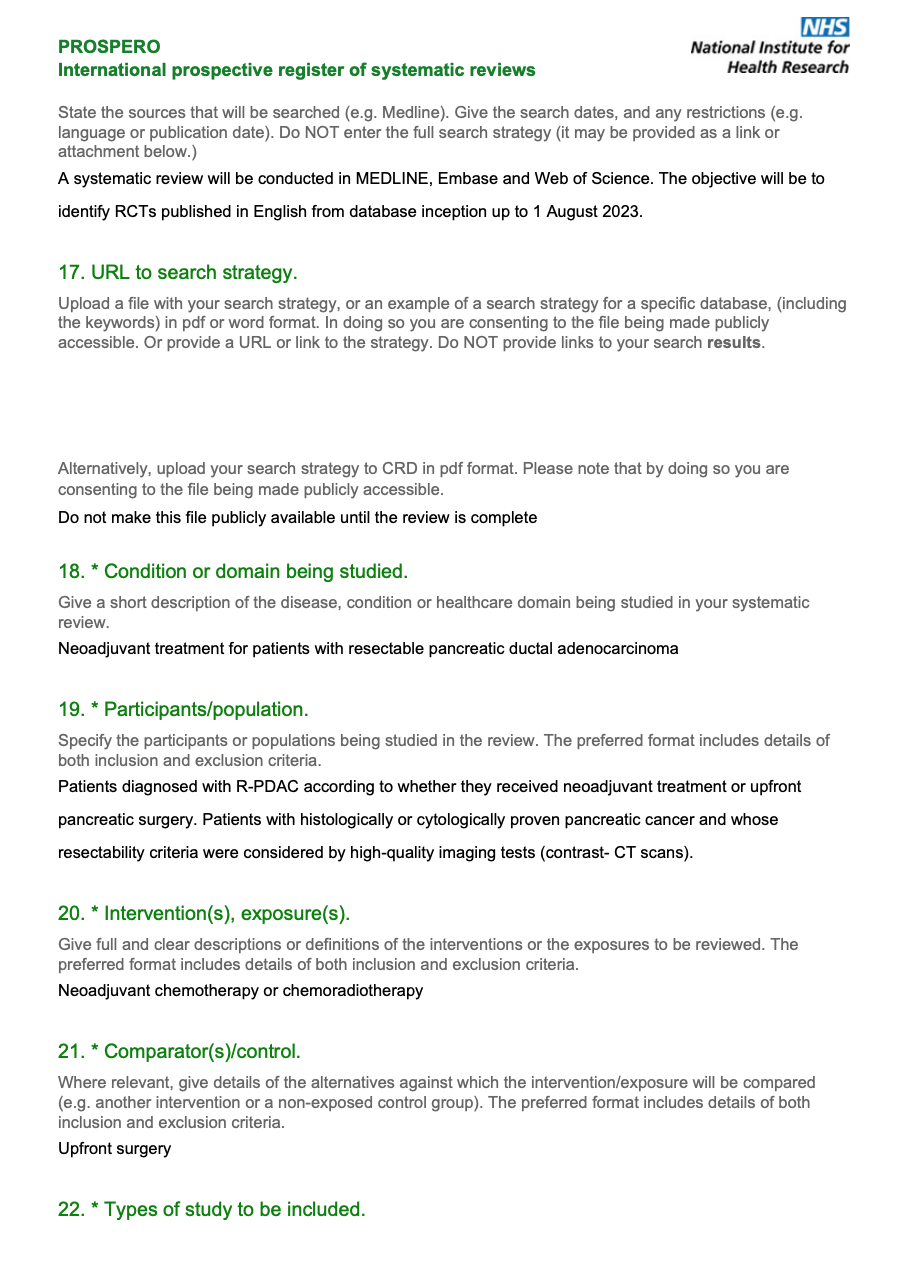


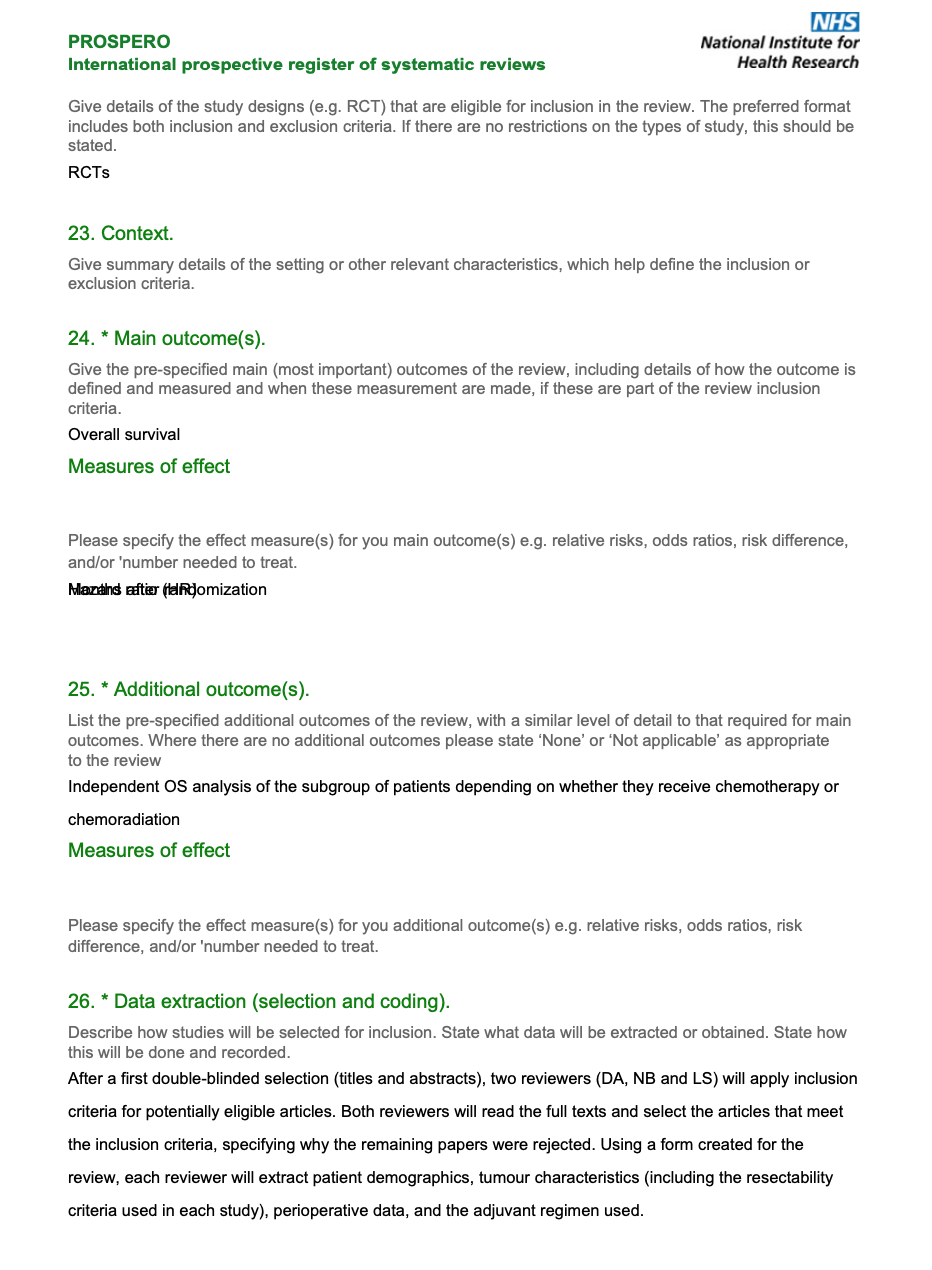


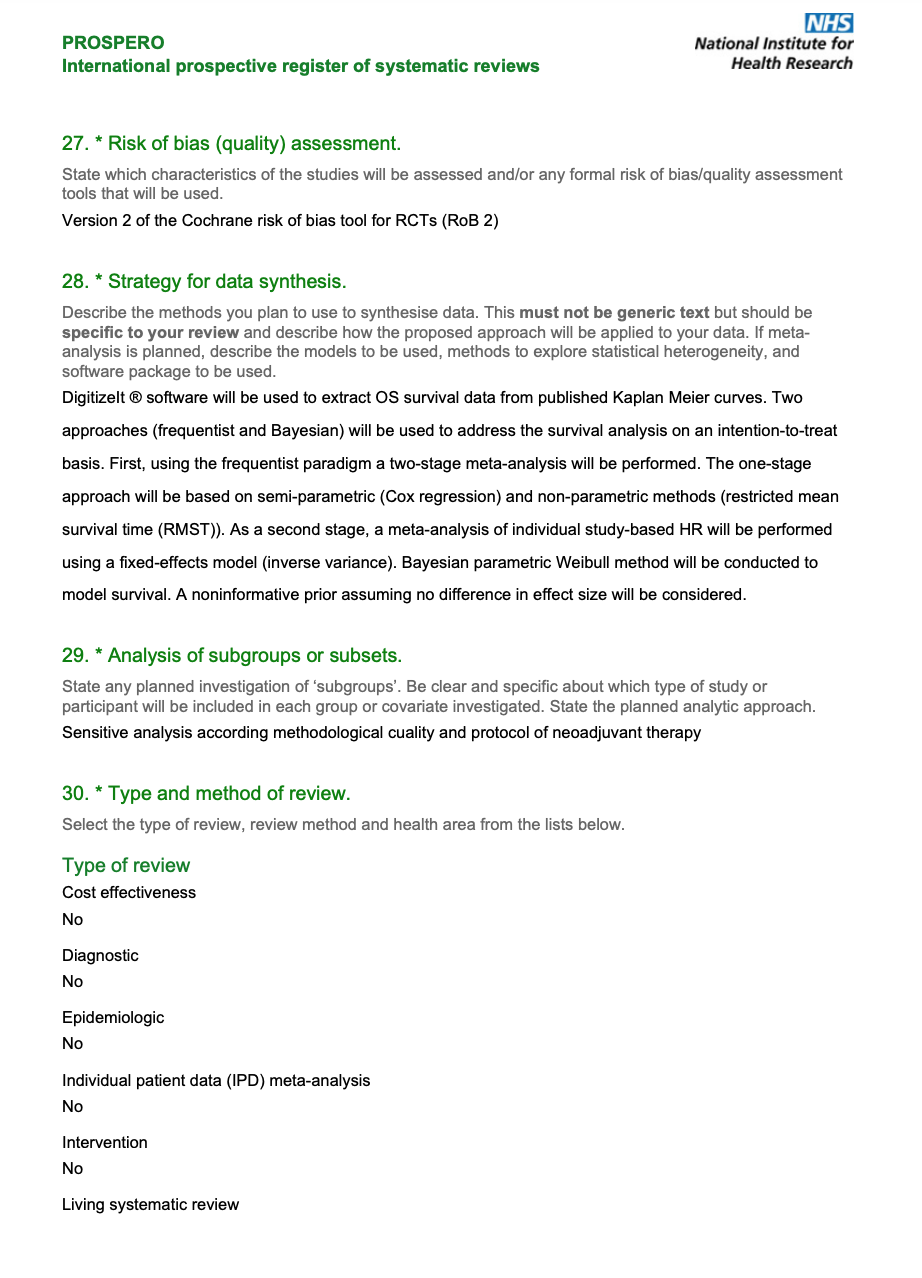


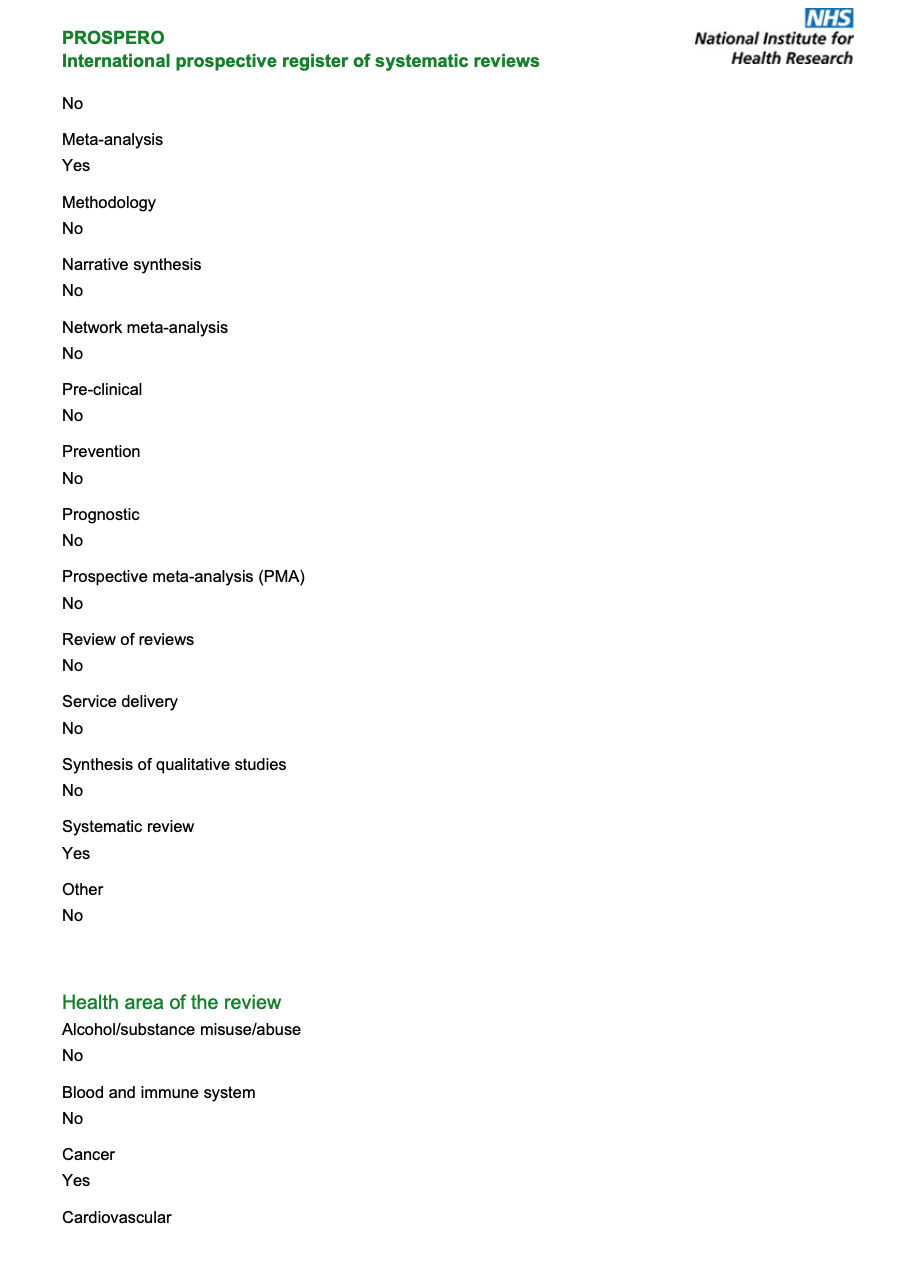


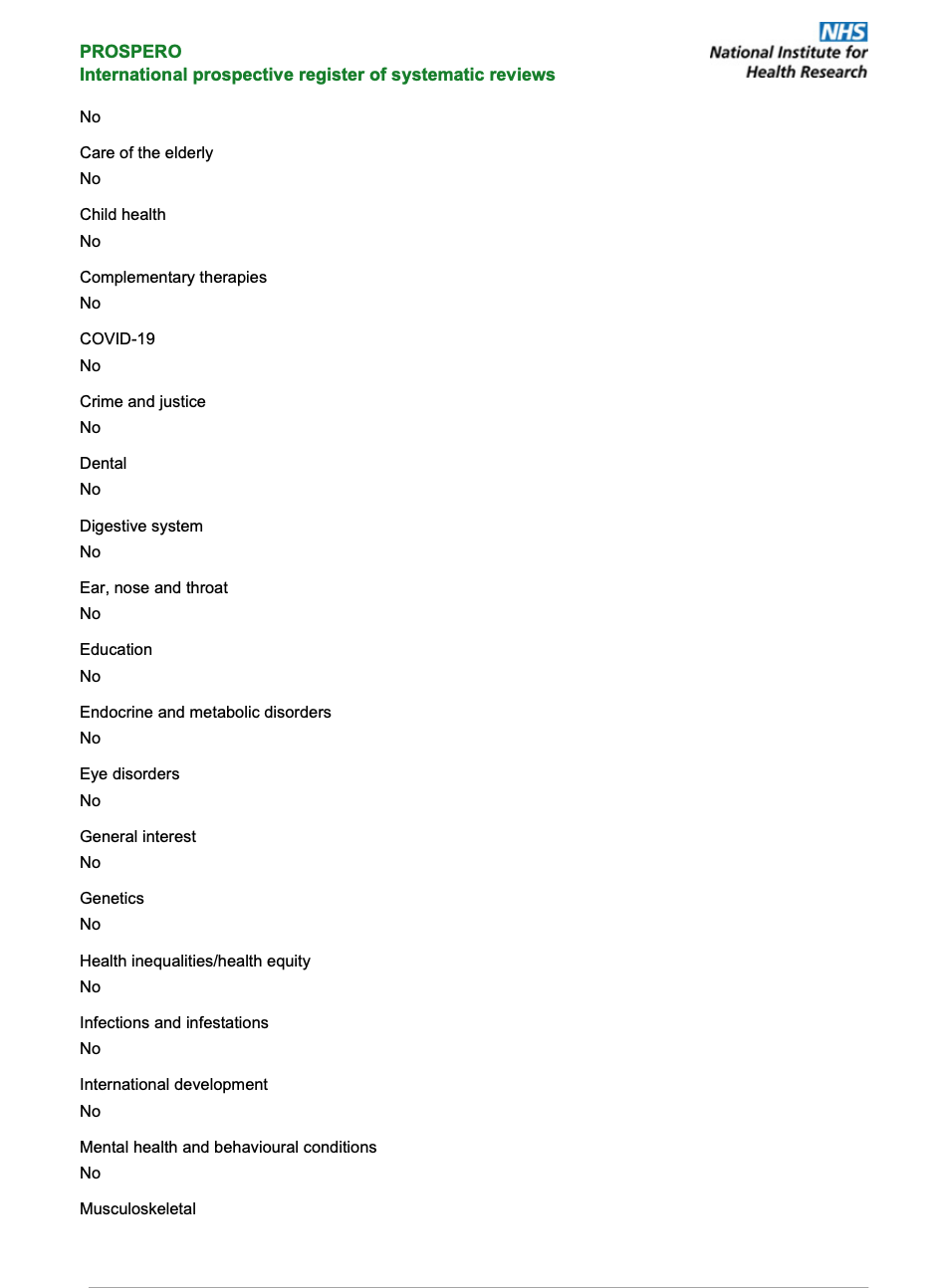


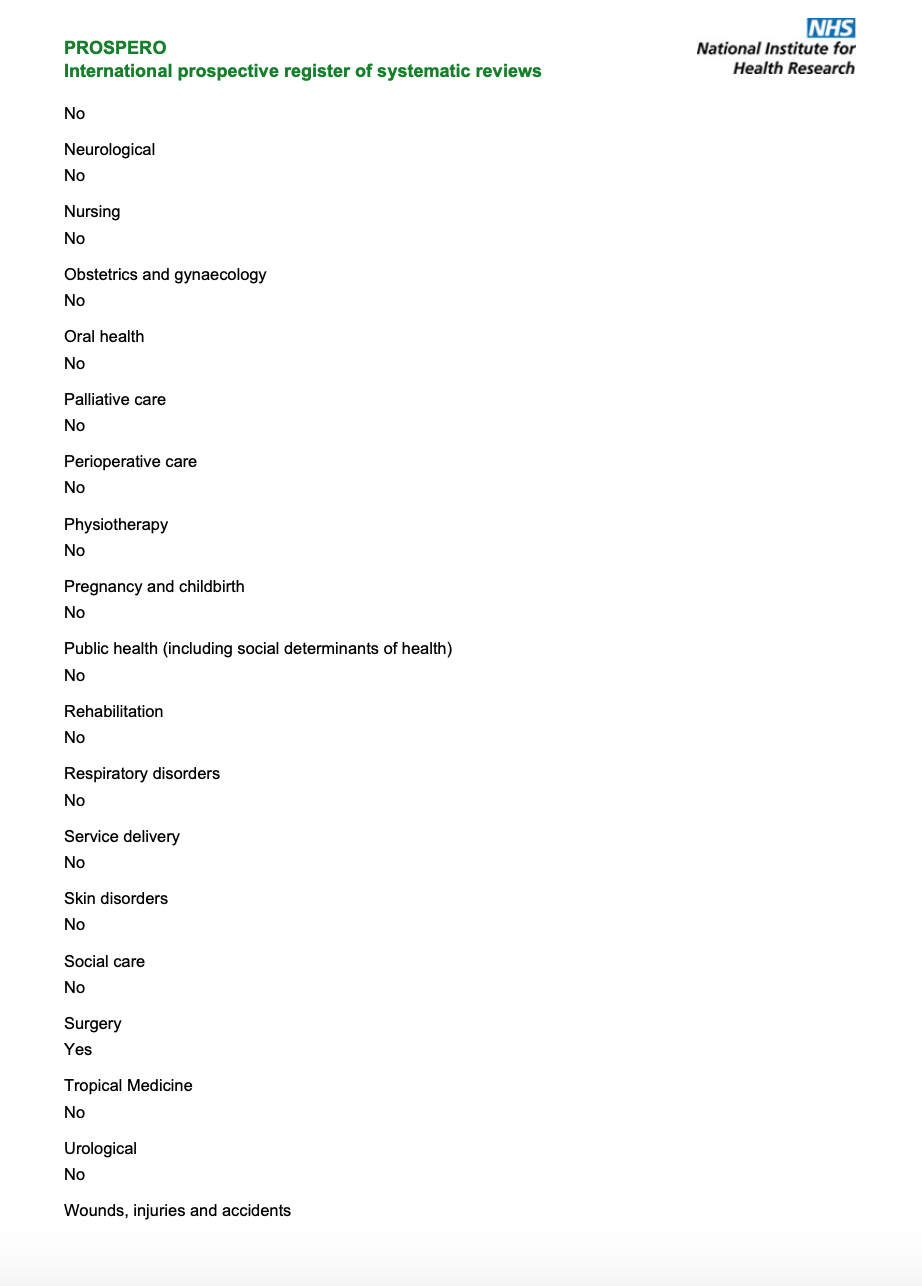


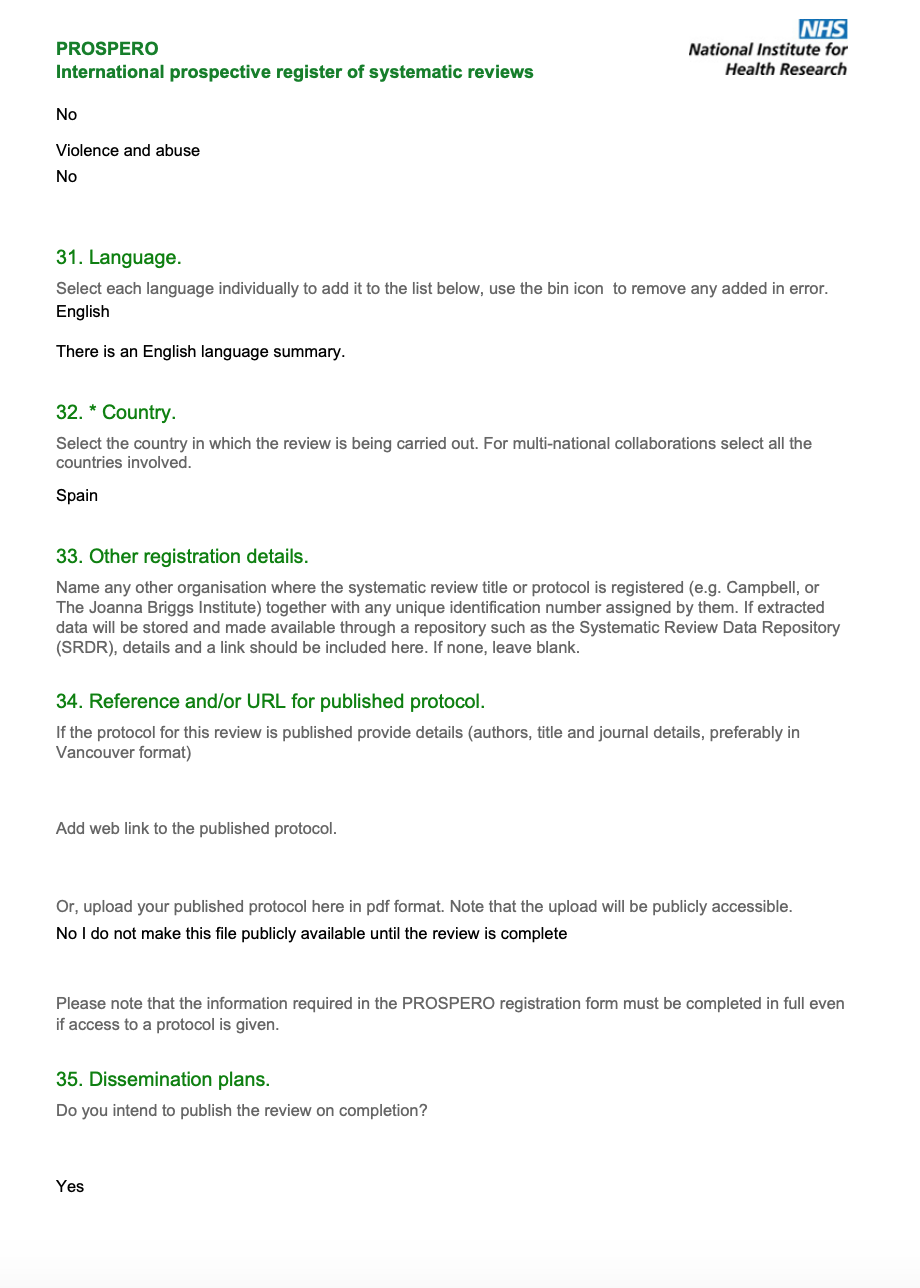


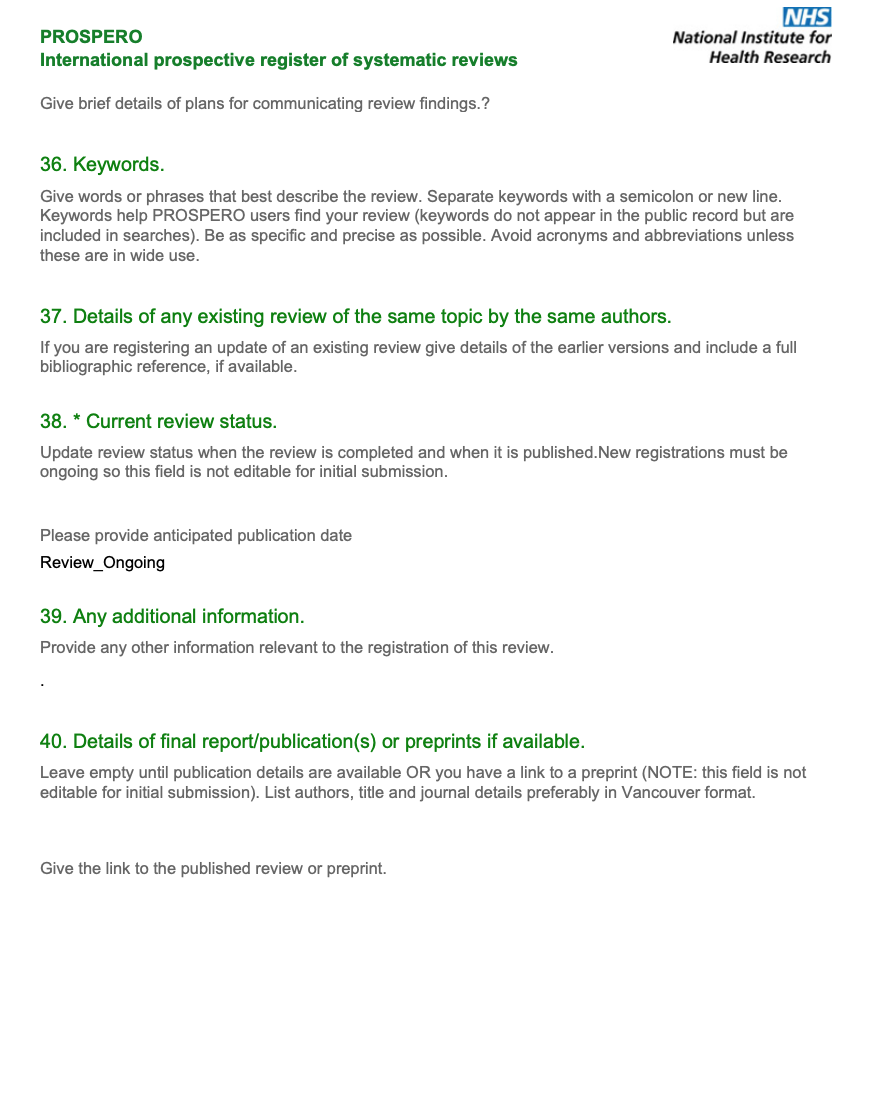


## PRISMA Checklist

| **Section/topic** | **#** | **Checklist item** | **Reported on page #** |
| --- | --- | --- | --- |
| **TITLE** | | |  |
| Title | 1 | Identify the report as a systematic review, meta-analysis, or both. |  |
| **ABSTRACT** | | |  |
| Structured summary | 2 | Provide a structured summary including, as applicable: background; objectives; data sources; study eligibility criteria, participants, and interventions; study appraisal and synthesis methods; results; limitations; conclusions and implications of key findings; systematic review registration number. |  |
| **INTRODUCTION** | | |  |
| Rationale | 3 | Describe the rationale for the review in the context of what is already known. |  |
| Objectives | 4 | Provide an explicit statement of questions being addressed with reference to participants, interventions, comparisons, outcomes, and study design (PICOS). |  |
| **METHODS** | | |  |
| Protocol and registration | 5 | Indicate if a review protocol exists, if and where it can be accessed (e.g., Web address), and, if available, provide registration information including registration number. |  |
| Eligibility criteria | 6 | Specify study characteristics (e.g., PICOS, length of follow-up) and report characteristics (e.g., years considered, language, publication status) used as criteria for eligibility, giving rationale. |  |
| Information sources | 7 | Describe all information sources (e.g., databases with dates of coverage, contact with study authors to identify additional studies) in the search and date last searched. |  |
| Search | 8 | Present full electronic search strategy for at least one database, including any limits used, such that it could be repeated. |  |
| Study selection | 9 | State the process for selecting studies (i.e., screening, eligibility, included in systematic review, and, if applicable, included in the meta-analysis). |  |
| Data collection process | 10 | Describe method of data extraction from reports (e.g., piloted forms, independently, in duplicate) and any processes for obtaining and confirming data from investigators. |  |
| Data items | 11 | List and define all variables for which data were sought (e.g., PICOS, funding sources) and any assumptions and simplifications made. |  |
| Risk of bias in individual studies | 12 | Describe methods used for assessing risk of bias of individual studies (including specification of whether this was done at the study or outcome level), and how this information is to be used in any data synthesis. |  |
| Summary measures | 13 | State the principal summary measures (e.g., risk ratio, difference in means). |  |
| Synthesis of results | 14 | Describe the methods of handling data and combining results of studies, if done, including measures of consistency (e.g., I^2^) for each meta-analysis. |  |

| **Section/topic** | **#** | **Checklist item** | **Reported on page #** |
| --- | --- | --- | --- |
| Risk of bias across studies | 15 | Specify any assessment of risk of bias that may affect the cumulative evidence (e.g., publication bias, selective reporting within studies). |  |
| Additional analyses | 16 | Describe methods of additional analyses (e.g., sensitivity or subgroup analyses, meta-regression), if done, indicating which were pre-specified. |  |
| **RESULTS** | | |  |
| Study selection | 17 | Give numbers of studies screened, assessed for eligibility, and included in the review, with reasons for exclusions at each stage, ideally with a flow diagram. |  |
| Study characteristics | 18 | For each study, present characteristics for which data were extracted (e.g., study size, PICOS, follow-up period) and provide the citations. |  |
| Risk of bias within studies | 19 | Present data on risk of bias of each study and, if available, any outcome level assessment (see item 12). |  |
| Results of individual studies | 20 | For all outcomes considered (benefits or harms), present, for each study: (a) simple summary data for each intervention group (b) effect estimates and confidence intervals, ideally with a forest plot. |  |
| Synthesis of results | 21 | Present results of each meta-analysis done, including confidence intervals and measures of consistency. |  |
| Risk of bias across studies | 22 | Present results of any assessment of risk of bias across studies (see Item 15). |  |
| Additional analysis | 23 | Give results of additional analyses, if done (e.g., sensitivity or subgroup analyses, meta-regression [see Item 16]). |  |
| **DISCUSSION** | | |  |
| Summary of evidence | 24 | Summarize the main findings including the strength of evidence for each main outcome; consider their relevance to key groups (e.g., healthcare providers, users, and policy makers). |  |
| Limitations | 25 | Discuss limitations at study and outcome level (e.g., risk of bias), and at review-level (e.g., incomplete retrieval of identified research, reporting bias). |  |
| Conclusions | 26 | Provide a general interpretation of the results in the context of other evidence, and implications for future research. |  |
| **FUNDING** | | |  |
| Funding | 27 | Describe sources of funding for the systematic review and other support (e.g., supply of data); role of funders for the systematic review. |  |

*From:*  Moher D, Liberati A, Tetzlaff J, Altman DG, The PRISMA Group (2009). Preferred Reporting Items for Systematic Reviews and Meta-Analyses: The PRISMA Statement. PLoS Med 6(7): e1000097. doi:10.1371/journal.pmed10000

# REFERENCES

1 Labori KJ, Bratlie SO, Biörserud C, Björnsson B, Bringeland E, Elander N, *et al.* Short-course neoadjuvant FOLFIRINOX versus upfront surgery for resectable pancreatic head cancer: A multicenter randomized phase-II trial (NORPACT-1). *J Clin Oncol*. 2023; **41**: LBA4005–LBA4005.

2 Versteijne E, Dam JL van, Suker M, Janssen QP, Groothuis K, Akkermans-Vogelaar JM, *et al.* Neoadjuvant Chemoradiotherapy Versus Upfront Surgery for Resectable and Borderline Resectable Pancreatic Cancer: Long-Term Results of the Dutch Randomized PREOPANC Trial. *J Clin Oncol*. 2022; **40**: 1220–1230.

3 Seufferlein T, Uhl W, Kornmann M, Algül H, Friess H, König A, *et al.* Perioperative or only adjuvant gemcitabine plus nab-paclitaxel for resectable pancreatic cancer (NEONAX)—a randomized phase II trial of the AIO pancreatic cancer group. *Ann Oncol*. 2023; **34**: 91–100.

4 Schwarz L, Bachet J-B, Meurisse A, Bouché O, Assenat E, Piessen G, *et al.* Resectable pancreatic adenocarcinoma neo-adjuvant FOLF(IRIN)OX-based chemotherapy: A multicenter, non-comparative, randomized, phase II trial (PANACHE01-PRODIGE48 study). *J Clin Oncol*. 2022; **40**: 4134–4134.

5 Reni M, Balzano G, Zanon S, Zerbi A, Rimassa L, Castoldi R, *et al.* Safety and efficacy of preoperative or postoperative chemotherapy for resectable pancreatic adenocarcinoma (PACT-15): a randomised, open-label, phase 2–3 trial. *Lancet Gastroenterology Hepatology*. 2018; **3**: 413–423.

6 Gelman A, Rubin DB. Inference from Iterative Simulation Using Multiple Sequences. *Stat Sci*. 1992; **7**.
